# Protactinium and the intersection of actinide and transition metal chemistry

**DOI:** 10.1038/s41467-018-02972-z

**Published:** 2018-02-12

**Authors:** Richard E. Wilson, Stephanie De Sio, Valérie Vallet

**Affiliations:** 1 Chemical Sciences and Engineering Division, Argonne National Laboratory, 9700 South Cass Avenue, Argonne, IL 60439 USA; 2 Université de Lille, CNRS, UMR 8523 - PhLAM - Physique des Lasers Atomes et Molécules, F-59000 Lille, France

## Abstract

The role of the 5*f* and 6*d* orbitals in the chemistry of the actinide elements has been of considerable interest since their discovery and synthesis. Relativistic effects cause the energetics of the 5*f* and 6*d* orbitals to change as the actinide series is traversed left to right imparting a rich and complex chemistry. The 5*f* and 6*d* atomic states cross in energy at protactinium (Pa), making it a potential intersection between transition metal and actinide chemistries. Herein, we report the synthesis of a Pa-peroxo cluster, A_6_(Pa_4_O(O_2_)_6_F_12_) [A = Rb, Cs, (CH_3_)_4_N], formed in pursuit of an actinide polyoxometalate. Quantum chemical calculations at the density functional theory level demonstrate equal 5*f* and 6*d* orbital participation in the chemistry of Pa and increasing 5*f* orbital participation for the heavier actinides. Periodic changes in orbital character to the bonding in the early actinides highlights the influence of the 5*f* orbitals in their reactivity and chemical structure.

## Abstract (web summary)

The role of the 5*f* and 6*d* orbitals in the chemical bonding of the actinide elements remains debated. Here, the authors synthesize and study a tetranuclear protactinium peroxo cluster and demonstrate that protactinium represents an intersection of actinide (5*f*) and transition metal (6*d*) chemistries.

## Introduction

The early actinide elements, because of their 5*f* electrons, exhibit a chemical behavior that is intermediate to the diffuse and directional *d*-orbital based transition metal chemistry and the largely localized and electrostatic bonding interactions in the 4*f* lanthanide elements. Their unique chemistry originally frustrated the identification of the actinide elements as a second *f*-series and not a fourth transition metal series^1^. The complex chemistry encountered in the early actinide series is a result of the changing relative energetics of the 5*f* and 6*d* electronic states across the actinide series, the energetics of which change with increasing *Z*, a consequence of relativistic effects and the incomplete shielding of the 5*f* orbitals from the positive nuclear charge^2^. In the case of the actinide dioxides, AnO_2(*s*)_, these effects cause the 5*f* orbitals at Th (*Z* = 90) to be higher in energy than the 6*d*, but at U (*Z* = 92) the 5*f* orbitals become lower in energy than the 6*d*, having crossed at Pa (*Z* = 91)^3^. This periodic trend in electronic structure effects both the reactivity and structure of the actinide ions. For example, it has been demonstrated that relativistic effects, along with the changing actinide orbital energies are responsible for the change from the bent *cis*- (*d*-character) to the linear *trans*- (*f*-character) geometry in the dioxide ions of Th and U, respectively^4^. Participation of the 5*f* and 6*d* orbitals to the chemistry of the actinides has also been exploited in the formation of metal ligand multiple bonds in U^5-13^, and the stabilization of a rare Pu(II) complex^14^. The location of this crossover point in the early actinides is not necessarily invariant; the particular crossing of 5*f* and 6*d* orbital energies being dependent on both actinide oxidation state and the chosen ligand system.

Provided these prior observations, and the general scarcity of chemical studies dealing with protactinium, Pa is identified as a unique opportunity for studying the periodic properties of the early actinide elements with respect to the participation of the 5*f* and 6*d* orbitals in their chemistry and the resulting effects on their structure and bonding. Our approach uses a comparative approach, combining experimental and computational methods to investigate the intersection of 5*f* and 6*d* chemistries at protactinium and how its chemistry is related to the *d*-orbital based transition metals and the *f*-dominated chemistry of the heavier early actinides.

**Fig. 1:**
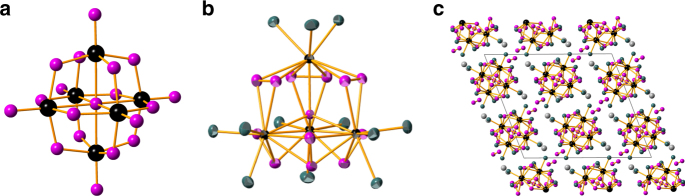
Transition metal and actinide clusters. Ball and stick representations of **a** the hexanuclear isopolyanion M_6_O_19_^8−^. **b** Thermal ellipsoid plot of the anionic cluster [Pa_4_O(O_2_)_6_F_12_]^2–^ (ellipsoids at 50% probability). **c** Packing diagram for Rb_6_[Pa_4_O(O_2_)_6_F_12_]∙(H_2_O_2_)_4_. The metal sites (Nb or Pa) are in black, oxygen atoms in magenta, fluorine in turquoise, and rubidium in gray

Protactinium’s transition metal homologues are Nb and Ta, all three sharing a closed shell pentavalent oxidation state. Niobium and tantalum are amphoteric metals as demonstrated by their ability to form both acid soluble complexes and alkaline soluble hexametalates, the simplest of these is the Lindqvist ion of the form M_6_O_19_^8−^ (M = Nb, Ta, Mo, W), Fig. 1a^15^. The transition metal hexametalates are composed of edge-sharing metal oxide octahedra with short M≡O terminal bonds, and symmetric metal–oxygen-metal bridges. For the actinides, the hexametalate of U(V) reported by Duval et. al. most closely resembles the hexametalates encountered in the Group V and VI transition metals^16^. Though it shares similarities in structure to its Mo(VI) and W(VI) homologues, it is principally composed of linear dioxo-cations of U(V), a molecular geometry known to be a hallmark of 5*f* participation in the formation of uranium oxygen multiple bonds^17^.

The linear dioxo-cation geometry common to the actinides, an indicator of 5*f* participation in the bonding, has not been observed for Pa(V) in aqueous systems and has been demonstrated to be exceptionally reactive in gas phase experiments, much more so than U, Np, and Pu because of the increasing participation of the 5*f* orbitals in the formation of the linear dioxo-bond for U, Np and Pu, compared to a small 5*f* contribution at Pa^18–21^. For Pa, the PaO^3+^ mono-oxo moiety has been observed crystallographically and spectroscopically, akin to the transition metal mono-oxo cations observed for Nb and Ta^20,22^. The proximity in energy of the *d*- and *f*-states at Pa and its geometric preference for a mono-oxo bond like that of the transition metals leads us to hypothesize that protactinium may be a candidate for forming a hexametalate complex of the Lindqvist type, M_6_O_19_^8−^, providing insight into the roles of the 5*f* and 6*d* orbitals at Pa, protactinium’s differences from its transition metal homologues, and further insight into the influence of the *f*-orbitals and electrons in the chemistry of the actinide elements. To this end, we undertook an experimental and computational study of the synthesis of a protactinium polyoxometalate.

Using an approach combining synthetic inorganic chemistry and quantum chemical calculations to explore the chemistry of the Group V hexametalates, M_6_O_19_^8−^ as exemplars of classic transition metal chemistry, and applying this approach to their 5*f* counterparts in Pa and U chemistry, we demonstrate that Pa represents an intersection between transition metal and actinide chemistry. We have synthesized a new protactinium peroxo compound [Pa_4_O(O_2_)_6_F_12_]^6−^ and determined it structure using single crystal X-ray diffraction. Quantum chemical calculations using both density functional theory (DFT) and the quantum theory of atoms in molecules (QTAIM) approach on our newly synthesized compound, and its actinide and transition metal homologues show Pa to be an important transition point for understanding the fundamental periodic properties of the early actinide series, specifically the importance and consequences of the changing participation of both the 5*f* and 6*d* orbitals in the chemistry of the early actinide elements.

## Results

### Synthesis and structure of [Pa_4_O(O_2_)_6_F_12_]^6−^

**Fig. 2:**
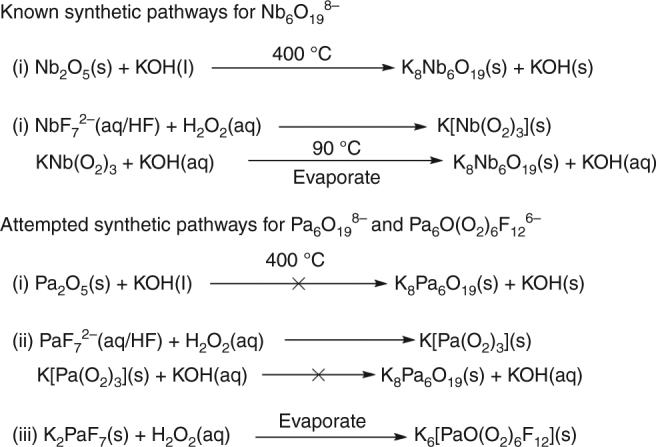
Reaction pathways to produce polyoxometalates. The known reactions for the synthesis of polyoxometalates are presented along with the reactions attempted for protactinium

The synthesis of Nb and Ta hexametalates can be approached from a variety of avenues including alkali hydroxide melts, room temperature dissolution of amorphous Nb or Ta oxide in base, or by decomposition of soluble peroxides in warm alkaline aqueous solutions^15,23,24^. All of these previously reported synthetic methods were tested with Pa(V) and all failed to produce the desired Pa_6_O_19_^6−^ polyoxometalate. The attempted reactions are summarized in Fig. 2.

Attempts to prepare a soluble protactinium–peroxo complex from Pa solutions in hydrofluoric and hydrochloric acid solutions by the addition of aqueous hydrogen peroxide resulted in the instantaneous precipitation of a yellow solid with significant effervescence of the solution. This solid is likely the unstable peroxide previously identified by Muxart^25^. The solid was then collected by centrifugation and subsequently dissolved in 2 M aqueous alkali hydroxide. The solubility of this Pa solid followed the trend Cs^+^ > Rb^+^ > K^+^ in agreement with previous reports of alkaline soluble Pa–peroxo complexes^25^. These alkaline solutions were effervescent and unstable, with a white solid precipitating after a few hours; the precipitate has been previously characterized as APa(O_2_)_3_ (A: K^+^, Rb^+^, Cs^+^)^25^. In our hands this precipitate could be redissolved with addition of excess aqueous hydrogen peroxide to the alkaline solution, further indicating its identity as a peroxo complex. No useful structural, spectroscopic, or stoichiometric data were able to be obtained from these solutions.

Encouraged by the apparent solubility of these Pa–peroxo complexes in alkaline solution and the known chemistry of Ta and Nb peroxides as precursors to the hexametalates, we experimented with the conditions for synthesizing a Pa peroxide precursor to the hexametalate. Direct dissolution of the heptafluoro salts of K_2_MF_7_ (M: Nb, Ta) in 30% wt H_2_O_2_ produces the known potassium tetraperoxo salts upon evaporation^26^. Similar reactions with the salts M_2_PaF_7_ (M: K^+^, Me_4_N^+^, Rb^+^, Cs^+^) demonstrated that all of these heptafluoro salts were soluble in 30% wt H_2_O_2(aq)_ except for the potassium salt. While it was expected that evaporation of these solutions would yield either the tetraperoxo complexes or the hexanuclear Pa_6_O_19_^8−^, single crystal X-ray diffraction data revealed the crystallized product to contain a tetranuclear protactinium cluster [Pa_4_O(O_2_)_6_F_12_]^6−^ shown in Fig. 1b,c. These anionic clusters co-crystallize with charge compensating cations, water, hydrogen peroxide and fluoride: Rb_6_[Pa_4_O(O_2_)_6_F_12_]∙(H_2_O_2_)_4_, Cs_6_[Pa_4_O(O_2_)_6_F_12_]∙(H_2_O_2_)_4_(H_2_O)_6_, ((CH_3_)_4_N)_7_[Pa_4_O(O_2_)_6_F_12_]∙(H_2_O_2_)(H_2_O)_14_F.

The observed cluster is composed of four protactinium(V) ions arranged at the vertices of a slightly distorted tetrahedron, Fig. 1b. At the center of the tetrahedron is a µ_4_–oxygen atom coordinating all four Pa ions, the surety of its identity as oxygen and not fluoride is provided by the charge balancing cations external to the main molecular species in the three different cluster structures (Me_4_N^+^, Rb^+^, Cs^+^). The vertices of the tetrahedron are linked to each other by edge-sharing µ_2_:η^2^-peroxo bridges. The protactinium coordination sphere is completed by three fluoride ions capping each of the protactinium ions. The coordination number of each protactinium ion is ten, the first such observed definitively in a Pa complex^27^, but not an uncommon coordination number for actinide ions whose coordination numbers have been reported as high as 16^28^. All bond distances and angles are presented in Supplementary Figures 1–3, and Supplementary Tables 1–6 for all of the crystal structures determined. Table 1 summarizes the structural metrics for the Rb^+^ salt.

**Table 1:** Bond distances and angles for Rb_6_[Pa_4_O(O_2_)_6_F_12_](H_2_O_2_)_4_

**Bond**	**Distance (Å)**
Pa(1)–µ_4_O	2.311(3)
Pa(2)–µ_4_O	2.315(3)
Pa(1)–O(peroxo)	2.343(4), 2.360(4)
	2.359(4), 2.376(4)
	2.386(4), 2.390(4)
Pa(2)–O(peroxo)	2.328(4), 2.340(4)
	2.343(4), 2.372(4)
	2.384(4), 2.393(4)
Mean Pa–(peroxo)	2.36(2)
Pa(1)–F	2.160(3), 2.155(3)
	2.229(3)
Pa(2)–F	2.155(3), 2.169(3)
	2.247(3)
Mean Pa–F	2.19(4)
Pa–Pa	3.762(1) x 2
	3.778(3) x 2
	3.779(3) x 2
mean Pa–OO–Pa °	106.0(3)°
mean Pa–µ_4_O–Pa °	109.5(3)°

The central tetrahedral oxygen atom bonds with an average Pa–O bond distance of 2.313(3) Å, and a mean bond angle Pa–O–Pa of 109.5(3)°, making it a near perfect tetrahedron. The Pa peroxo linkages span 2.328(4)–2.393(4) Å with a mean of 2.36(2) Å, in agreement with previously reported actinide peroxo bond distances^29^. The Pa–Pa distances average 3.77(1) Å, in comparison to the Pa–Pa distances in tetravalent PaO_2_ of 3.57 Å, and 3.22 Å in the tetragonal phase of protactinium metal^30^. The charge of the [Pa_4_O(O_2_)_6_F_12_]^6–^ anion is compensated by co-crystallized cations in the crystal interstices, Fig. 1c.

Within the actinide peroxide literature, this structural motif is unique, the majority of the observed actinide peroxide structures taking on significant curvature as a result of both the peroxide ligand, the actinyl oxygen, and more distant interactions with charge compensating cations in these complexes forming nanospheres, belts, and in some cases fullerene-like connectivities to name only a few^29^. The formation of discrete tetranuclear metal clusters about a tetrahedral oxygen atom are rare for the actinides with few examples known to date^31^. However, a variety of tetranuclear Ce(IV) complexes with a tetrahedral motif are known^32,33^.

### Quantum chemical calculations

Quantum chemical calculations at the density functional theory (DFT) level were performed on the synthesized Pa peroxide cluster and the properties of the molecular bonding analyzed using Bader’s quantum theory of atoms in molecules (QTAIM) approach^34^. In order to preserve the tetrahedral symmetry observed experimentally at the central µ_4_–O atom, the overall charge of the cluster as computed is 2− (four charge compensating alkali metal cations are included in the calculation), in contrast to the 6− charge observed experimentally for the isolated cluster unit and its six charge compensating cations. Tetrahedral symmetry at the μ_4_–O cannot be achieved computationally if all six charge compensating cations are included in the model. The corresponding U(V) cluster was also calculated for comparison and to provide insight into the periodic properties of the *f*-electrons in this class of molecules.

The optimized geometries for the computed Pa(V) and U(V) structures are presented in Table 2, and are in excellent agreement with the experimental results for the reported Pa cluster, Table 1, with the exception that the inclusion of only four of the six charge compensating cations results in slightly longer Pa–F bond distances from that observed experimentally, likely because of the interaction between the fluoride ions and the included cations outside the molecular cluster. To minimize errors introduced because of the change in the calculated versus experimental charge of the system, both the Pa(V) and U(V) clusters were calculated identically allowing for comparison of their overall bonding properties using the calculated QTAIM parameters shown in Table 2.

**Table 2:** Average bond distances and QTAIM characteristics of the bond critical points in Rb_4_[An_4_O(O_2_)_6_F_12_]^2^^–^ and Cs_4_[An_4_O(O_2_)_6_F_12_]^2^^–^ (An = Pa(V), U(V))

Bond	QC	Rb_4_[Pa_4_O(O_2_)_6_F_12_]^2−^	Cs_4_[Pa_4_O(O_2_)_6_F_12_]^2−^	Rb_4_[U_4_O(O_2_)_6_F_12_]^2−^	Cs_4_[U_4_O(O_2_)_6_F_12_]^2−^
An–μ_4_O	r(M–μ_4_O)	2.288	2.286	2.267–2.276	2.265–2.270
	*ρ*_b_	0.09	0.09	0.09	0.09
	∇^2^*ρ*_b_	0.24	0.24	0.27	0.27
	DI(M–L)	0.45	0.45	0.46	0.46
An–O(peroxo)	r(M–O)	2.336	2.336	2.293–2.358	2.291–2.357
	*ρ*_b_	0.08	0.08	0.07–0.09	0.07–0.09
	∇^2^*ρ*_b_	0.22	0.22	0.22–0.24	0.22–0.24
	DI(M–L)	0.43	0.43	0.46–0.50	0.46–0.50
An–F	r(M–F)	2.256	2.254	2.226–2.251	2.223–2.255
	*ρ*_b_	0.09	0.09	0.09	0.09
	∇^2^*ρ*_b_	0.30	0.30	0.31	0.31
	DI(M–L)	0.47	0.47	0.50	0.50
An–An		3.735	3.732	3.703–3.718	3.700–3.714
*Bond angles*					
An–μ_4_O–An		109.5	109.5	109.3	109.5
An–OO–An		106.1	106.1	105.7	106.1

Bond distances in Å, angles in degrees, *ρ*_b_ and ∇^2^*ρ*_b_ are the electron density and the Laplacian at the BCP given in e^−^ bohr^–3^ and e^−^ bohr^−5^, respectively

DI(ML) is the delocalization index

**Fig. 3:**
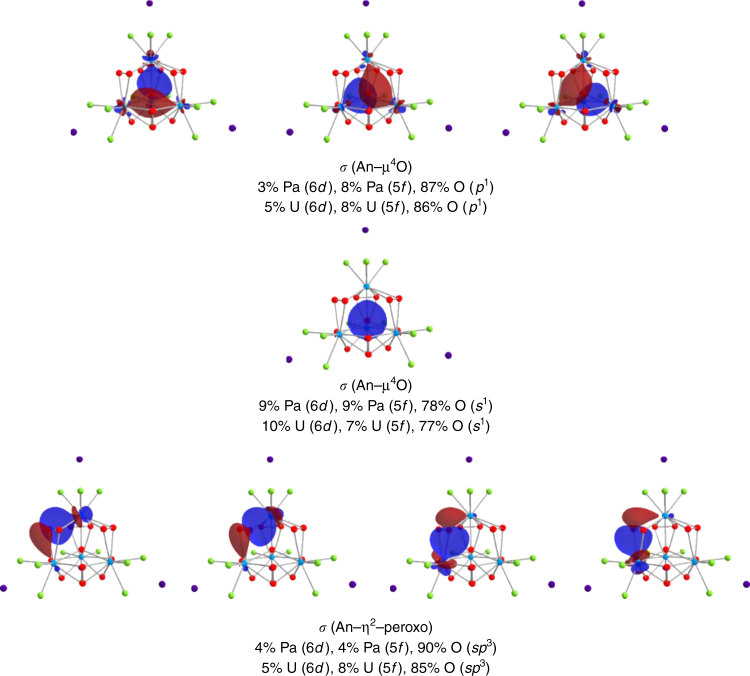
Plots of the natural localized molecular orbitals of Cs_4_[An_4_O(O_2_)_6_F_12_]^2−^. This figure shows the four *σ* bonds to the central μ_4_–oxygen, the four *σ*-bonds between the actinide and the η^2^–peroxo bond. The atomic orbital contributions to the localized natural molecular orbitals are provided and highlight the increasing 5*f* participation in moving from Pa to U. The isosurface cutoff is 0.03. The metal atoms are turquoise spheres, the oxygen in red, fluorine in green, and the rubidium atoms in purple

Because Pa is known to form PaO^3+^ mono-oxo bonds and not the linear dioxo bonds common to the heavier actinides, it was desirable to understand if the Pa–µ_4_O bond had any multiple bond character. The results of our DFT calculations and the QTAIM analysis of the results indicates that the Pa–µ_4_O bond is ionic in nature consisting of a small ~11% contribution from the protactinium based 5*f* (8%) and 6*d* (3%) orbitals and is principally oxygen 2*p* (87%) based as shown in Fig. 3 and Table 2. The orbitals of the central µ_4_–oxygen are not *sp*^3^ hybridized despite its tetrahedral symmetry and can be thought of as O^2−^ with its filled octet. The same is true for the U(V) cluster, except that there is marginally greater metal character in the U–O interaction. The Pa–μ_4_O bond is unlike the short uranium oxygen bonds formed in the cluster reported by Duval and does not show any multiple bonding character as would be expected for a protactinium mono-oxo bond^16^. This is not surprising provided the relatively long Pa–O bond distance of 2.31 Å, versus an expected 1.9 Å for a multiply bonded Pa–O bond^20^. Similar to the Pa–μ_4_O bonds, the QTAIM metrics indicate that the Pa–peroxo bonds are electrostatic in nature, with nearly equal contributions from the Pa 5*f* (4%) and 6*d* (4%), but overwhelmingly O 2*p* (90%) in character. Importantly, the QTAIM and natural molecular orbital calculations for U(V) highlight increased metal character to the bonds in the uranium cluster, particularly with respect to the metal-peroxide bond. The increasing contributions from the actinide 5*f* and 6*d* orbitals to all of the An–O bonds underscores the changing relative energetics of the 5*f* and 6*d* orbitals as one moves from Pa to U, demonstrating the periodic stabilization of the 5*f* orbitals in the early actinides and their increased participation in the formation of chemical bonds. The QTAIM metrics in Table 2 also show that, with respect to the electron density at the bond critical point and the Laplacian at the bond critical point, there is no accumulation of charge between the actinide and its ligands, which indicates that there is limited orbital overlap between either metal 5*f* and 6*d* and the oxygen or fluorine 2*p* orbitals, despite the increase in metal character to the bonding. Kerridge has recently demonstrated that in the absence of orbital overlap, the delocalization index can be used as an indicator of metal–ligand orbital energy matching, or so called ‘degeneracy-driven covalency’^35^. In our calculations, there are modest increases in the delocalization index between the Pa and U peroxo-clusters that may indicate a trend towards increased degeneracy-driven covalence as one moves to heavier 5*f* elements, consistent with the periodic stabilization of the 5*f* orbital energies across the actinide series.

### Transition metal peroxo-clusters

Both experimental efforts and computational efforts failed to produce a transition metal homologue of the reported tetranuclear peroxo cluster described above. In computations where Ta or Nb were substituted for Pa in Rb_4_[Pa_4_O(O_2_)_6_F_12_]^2−^ the transition metal–peroxo bonds as well as the entire cluster was found to be unstable. The instability of the transition metal clusters is likely because the cluster is formally ten coordinate, thus requiring 20 electrons to form 10 two-electron sigma bonds, necessitating one additional metal based orbital than the nine available; the actinides have the *f*-orbitals available for bond formation and therefore can accommodate the higher coordination number. Undeterred, we then considered the formation of a Lindqvist type hexametalate of the form Pa_6_O_19_^8−^, and how its chemistry would compare to the transition metals and the heavier early actinides.

### Actinide and transition metal hexametalates

To determine the feasibility of forming an actinide hexametalate we calculated the reaction energetics for the formation of the actinide and transition metal hexametalates via the hydrolysis of the pentachlorides of Nb, Ta, Pa, and U, using the reaction presented^24^.

1 $${\mathrm{6MCl}}_{5} + {\mathrm{38OH}}^{-} \to {\mathrm{M}}_{\mathrm{6}}{\mathrm{O}}_{\mathrm{19}}^{8-} + {\mathrm{30Cl}}^{-} + {\mathrm{19H}}_{\mathrm{2}}{\mathrm{O}}$$

The reaction free energies were calculated using a continuum model for water and are presented relative to the Nb reaction energy, Table 3. The absolute values of the reaction energies indicate that all of the reactions are favorable; however, the trend in reaction energies is more reliably discussed than the absolute value of the computed free energies. The trend in reaction energies shows that the transition metals, in agreement with their established chemistry, will form the hexametalates and though the reaction free energies are higher for Pa(V) and U(V), these actinide hexametalates are nevertheless stable in silico, albeit with different final structures.

**Table 3:** Metal–oxygen bond distances and QTAIM characteristics of the [M_6_O_19_]^8–^ clusters

Metal	**QC**	[Nb_6_O_19_]^8−^ (*O*_*h*_)	[Ta_6_O_19_]^8−^ (*O*_*h*_)	[Pa_6_O_19_]^8−^ (*O*_*h*_)	[U^V^_6_O_19_]^8−^ (*O*_*h*_, imag)	[U^V^_6_O_19_]^8−^ (*T*_*h*_)	[U^VI^_6_O_19_]^2−^ (*O*_*h*_, imag)	[U^VI^_6_O_19_]^2−^ (*T*_*h*_)
M–μ_6_O	r(M–O)	2.419	2.412	2.664	2.621	2.626	2.568	2.596
	*ρ*_b_	0.04	0.05	0.04		0.04		0.05
	∇^2^*ρ*_b_	0.16	0.17	0.11		0.12		0.13
	DI(M–L)	0.19	0.19	0.27		0.27		0.29
M–μ_2_O	r(M–O)	1.993	1.998	2.183	2.160	2.254/2.079	2.092	2.328/1.919
	*ρ*_b_	0.13	0.13	0.11		0.09/0.14		0.07/0.22
	∇^2^*ρ*_b_	0.42	0.48	0.31		0.28/0.36		0.25/0.40
	DI(M–L)	0.68	0.65	0.78		0.55/1.03		0.42/1.49
M–O_yl_	r(M–O)	1.861	1.861	2.072	2.037	2.040	1.827	1.836
	*ρ*_b_	0.17	0.18	0.15		0.16		0.27
	∇^2^*ρ*_b_	0.60	0.70	0.37		0.36		0.42
	DI(M–L)	1.11	1.09	1.17		1.36		1.87
Δ*G*_f_		0.0	–122.7	511.8	—	500.6	—	—

Bond distances in Å; electron density at the bond critical point, e^–^ bohr^–3^; the Laplacian at the bond critical point, e^–^ bohr^–5^; and the delocalization index are shown. Formation free energies computed in a water solvent in kJ mol^−1^ Δ*G*_f_ of [M_6_O_19_]^8−^ clusters according to the reaction: 6MCl_5_ + 38OH^−^ → [M_6_O_19_]^8−^ + 30Cl^−^ + 19H_2_O relative to Nb

**Fig. 4:**
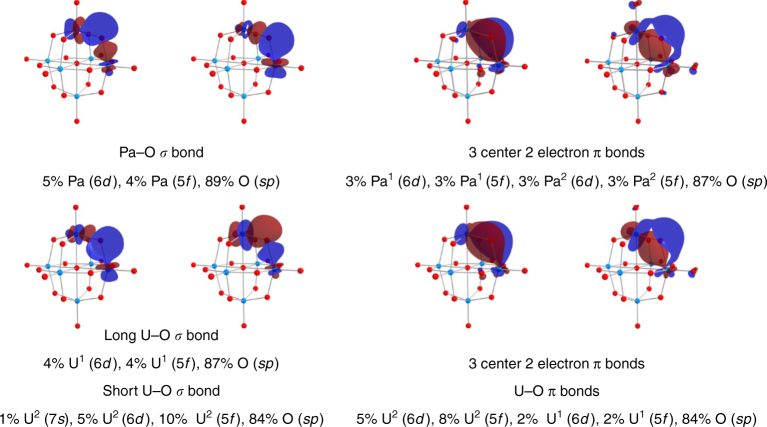
Plots of the natural localized molecular orbitals of the calculated Pa and U hexametalates. The oxidation state of the U(V) hexametalate was verified at the end of the calculation. The atomic orbital contributions to the localized natural molecular orbitals are provided. The isosurface cutoff is 0.03. The metal atoms are turquoise spheres and the oxygen in red

The optimized geometries of the calculated transition metal and actinide hexametalate species fall into two categories, *O*_*h*_ or *T*_*h*_ symmetry. The Nb, Ta, and Pa hexametalates are found to have *O*_*h*_ symmetry. The calculated U(V) and U(VI) hexametalate geometries distort forming species with *T*_*h*_ symmetry. Attempts to force the U complexes to octahedral symmetry resulted in unstable complexes as identified by imaginary vibrational frequencies for these anionic clusters as previously observed by Jiang et al. in their study of uranium hexametalates^36^. Inspection of the bond distances, QTAIM parameters, and the natural localized molecular orbitals in these molecules also reveals a break in the nature of the bonding between the transition metals and within the actinides, Table 3, and Fig. 4.

For the *O*_*h*_ hexametalates of Nb, Ta, and Pa, there are three unique bond distances, the short terminal M–O bond at the vertices of the octahedron, the bridging M–µ_2_–O–M bonds, and the considerably longer central µ_6_–O metal bonds. The short bonds to the terminal oxygen atoms have multiple bond character as shown by their QTAIM metrics. The QTAIM metrics indicate that the metal and the bridging oxygen bonds are also relatively electrostatic in nature as indicated by the lack of charge accumulation at the bond critical points. Interestingly however, the value of the Laplacian for the Pa hexametalate is smaller than that calculated for the transition metals and is accompanied by a larger delocalization index. Though tempting to attribute this to increased covalent bonding in the Pa hexametalate versus the transition metals, it could also be a consequence of the larger radial extent of the 5*f* and 6*d* orbitals in Pa vs. the less expansive 4*d* and 5*d* in Nb and Ta, respectively^36^. Similar behavior is observed in the metal bonds to the central oxygen atom, which by the QTAIM metrics all indicate a weak and ionic like metal–oxygen interaction and, in a similar manner to the central oxygen atom in the synthesized tetranuclear Pa–peroxo complex, can be thought of as an unhybridized O^2−^ atom.

In contrast to the structures with *O*_*h*_ symmetry calculated for Nb, Ta, and Pa, the U(V) and U(VI) hexametalates possess *T*_*h*_ symmetry. The reduction in symmetry from *O*_*h*_ to *T*_*h*_ is caused by the distortion of the U–µ_2_–O–U bond distances to a one-short-one-long arrangement, much like that observed by Duval et al^16^. The QTAIM metrics show that in the uranium case, the electron density at the bond critical point increases for the short U–O bridging bond accompanied by a large increase in its delocalization index. These changes indicate a trend toward multiple bond character and within the framework provided by Kerridge suggest that any increase in U–O bond covalency is likely due to increasing metal–ligand orbital degeneracy and not increased orbital overlap^35^.

It was proposed by Jiang that the cause of the asymmetry observed for the uranium clusters was due to weaker U(5*f*)–O(2*p*) interactions in comparison to the stronger Mo, W, Sg (*d*)–O(2*p*) interactions in the transition metals. This does not explain the observed *O*_*h*_ symmetry of the Pa hexametalate and its symmetric bridging metal oxygen bonds like those observed in Nb and Ta^36^. Because the 5*f* orbitals of Pa would are expected to be higher in energy than the U 5*f* orbitals based on relativistic periodic trends, we might expect greater 6*d*, or rather less 5*f* character in the Pa–O bonds vs. the U system. This apparent transition between Nb, Ta, and Pa with stable *O*_*h*_ geometries and equidistant M–µ_2_–O–M bond distances to the distorted *T*_*h*_ symmetry of U leads us to ask, what differences in the molecular electronic structure are responsible for the change in structure between the transition metals, the actinides, and importantly between Pa and U?

### Orbital contributions to the metal oxygen bonds

To answer this, we looked to the natural localized molecular orbitals and a population analysis of those orbitals that comprise the metal–oxygen interactions in the hexametalates, Figure 4, Table 4, and Supplementary Figures 4–6. The transition metal–oxygen bonds are necessarily *d*-based for the transition metal hexametalates. The terminal M–O bond is a triple bond formed by one *σ* and two *π* bonds, while the bridging M–O–M bond is composed of two discrete *σ* bonds between each transition metal atom and the µ_2_–O atom along with a three-center-two-electron *π* bond across the M–O–M linkage. The same analysis for Pa_6_O_19_^8^^−^ shows participation of both the 5*f* and 6*d* orbitals in the formation of the Pa–O bonds. The terminal Pa–O bond is formally a triple bond, the *σ* and two *π* contributions are of equal 5*f* (5%) and 6*d* (5%) character. The three-center-two-electron *π* bond in the µ_2_–O linkages identified in the transition metals is also present in the Pa hexametalate and is principally oxygen based with small contributions from both Pa 5*f* (3%) and 6*d* (3%) orbitals with the *σ* component to this bond partitioned Pa 5*f* (4%) 6*d* (5%).

**Table 4:** Atomic orbital contributions to the natural localized molecular orbitals in the in the [M_6_O_19_]^8–^ clusters

	*σ* (M–μ_2_O)	*π* (M–μ_2_O)
Nb(V)	12% Nb (4*d*), 86% O (*s*/*p*)	3C 7% Nb_1_ (4*d*), 7% Nb_2_ (4*d*), 86% O (*p*^1^)
Ta(V)	11% Ta (5*d*), 86% O (*s*/*p*)	3C 6% Ta_1_ (5*d*), 6% Ta_2_ (5*d*), 86% O (*p*^1^)
Pa(V)	5% Pa (6*d*), 4% Pa (5*f*), 89% O (*s*/*p*)	3C 3% Pa_1_ (6*d*), 3% Pa_1_ (5*f*), 3% Pa_2_ (6*d*), 3% Pa_2_ (5*f*), 87% O (*p*^1^)
U(V)	Short *σ*: 5% U (6*d*), 10% U (5*f*), 84% O (*s*/*p*)	3C 5% U_1_ (5*d*), 8% U_1_ (5*f*), 2% U_2_ (5*d*), 2% U_2_ (5*f*), 84% O (*p*^1^)
	Long *σ*: 1% U (7*s*), 5% U (6*d*), 4% U (5*f*), 87% O (*s*/*p*)	
U(VI)	Short *σ*: 5% U (6*d*), 12% U (5*f*), 81% O (*s*/*p*)	3C 5% U_1_ (5*d*), 10% U_1_ (5*f*), 2% U_2_ (5*d*), 2% U_2_ (5*f*), 81% O (*p*^1^)
	Long *σ*: 1% U (7*s*), 5% U (6*d*), 3% U (5*f*), 87% O (*s*/*p*)	
	*σ* (M–O_yl_)	*π* (M–O_yl_)
Nb(V)	2% Nb (5*s*), 13% Nb (4*d*), 83% O (*sp*^2^)	13% Nb (4*d*), 86% O (*p*^1^)
Ta(V)	3% Ta (6*s*), 12% Ta (5*d*), 83% O (*sp*^1^)	13% Ta (5*d*), 87% O (*p*^1^)
Pa(V)	5% Pa (6*d*), 5% Pa (5*f*), 87% O (*sp*^1^)	5% Pa (6*d*), 5% Pa (5*f*), 87% O (*p*^1^)
U(V)	6% U (6*d*), 10% U (5*f*), 83% O (*sp*^2^)	6% U (6*d*), 9% U (5*f*), 85% O (*p*^1^)
U(VI)	7% U (6*d*), 14% U (5*f*), 77% O (*sp*^2^)	6% U (6*d*), 14% U (5*f*), 79% O (*p*^1^)

3-center 2 electron bonds are flagged 3C

For the U-hexametalate, underlying the *T*_*h*_ symmetry are asymmetric bridging µ_2_–O linkages, one short and one long U–O bond within the three-center-two-electron bond of the U–O–U bridge. The shorter bond (1.892 Å) in the U–O–U bridge contains equal 5*f* and 6*d* contributions from the U atoms. The *π* components to this bond, the three–center-two-electron bond, contain contributions from two uranium atoms in the U–O–U bridge with increased *f*-character in the shorter portion of the bonds relative to those observed in the Pa complexes. The longer (2.348 Å) U–O *σ* bond in the bridge has more metal character than the short *σ* bond with nearly twice as much *f*-character (10% 5*f*) as *d*-character (4% 6*d*). Increases in the *f*-character to the molecular orbitals are also demonstrated in the short terminal triple bond for U_6_O_19_^8−^ underscoring the increasing participation of the 5*f* versus 6*d* as the metal is changed from Pa to U; the 5*f* orbital contribution to the bonding orbitals formed for U are nearly twice that of the 6*d* component, whereas at Pa they are nearly equal. The QTAIM metrics also support increased metal participation to the bonding as shown not only in the case of the U(V) hexametalates as discussed above, but also in the calculated U(VI) hexametalates, which is isoelectronic with Pa(V), where there are significant increases to both the electron density at the bond critical points accompanied by increases in the delocalization indices.

Inspection of the metal electron populations shows that for both the Pa and U clusters, the *d*-populations are equal, but the total *f*-population increases from Pa, where it is nearly equal that of the *d*-population, to U where a larger *f*-population is calculated. It is important to note that the uranium cluster is U(V), a 5*f*^1^ ion, and when we take this into account for the calculated electron populations we still see an increase in the 5*f* population in moving from Pa to U, Table 5. The significant involvement of the 5*f* orbitals is also observed for the hexavalent oxidation state. The enhanced *f*-population has been argued to be an indicator of increasing covalent character in the actinides attributed to the increasing participation of the *f*-orbitals in the formation of a chemical bond, though not necessarily increased orbital overlap^18,35,37,38^.

**Table 5:** Total metal valence *d-* and *f*-populations

	Nb(V)	Ta(V)	Pa(V)	U(V)	U(VI)
*n*(*f*)	0.02	0.01	1.37 (1.37)	2.64 (1.64)	2.56
*n*(*d*)	2.65	2.39	1.32	1.31	1.49

Numbers in parentheses reflect the difference to the formal occupations in oxidation state (V)

Both experiment and independent theory have demonstrated that the increased participation of the *f*-orbitals from Pa to Pu drives an increase in metal–oxygen covalent character through 5*f*-ligand orbital degeneracy in the early actinides. This has been shown to manifest itself as a decrease in reactivity with respect to exchange rates of the actinyl oxygen atoms about these ions and their ability to form metal–ligand multiple bonds^5,6,8,9,18,21,37^. The identification of this important periodic trend of increasing *f*-orbital and electron contributions to the molecular bonding serves to highlight the changing influence of the 5*f* electrons across the actinide series and how the unique electronic contributions of the 5*f* electrons and orbitals to the chemistry of the early actinide elements drives structure and bonding in these elements.

## Discussion

In an attempt to synthesize a polyoxometalate containing Pa, the actinide homologue of Nb and Ta, we instead synthesized a heretofore unknown peroxo-cluster of Pa, [Pa_6_O(O_2_)_6_F_12_]^6−^. Quantum chemical calculations and analysis of the bonding in this cluster, and the calculated Lindqvist type hexametalates, M_6_O_19_^8−^, containing Nb, Ta, Pa, and U were performed to understand the fundamental chemical differences in this class of molecules with respect to the participation of the 5*f* orbitals to the bonding in these systems. Participation of the *f*-orbitals in chemical bonding is unique to the actinide elements and is a consequence of the relativistic stabilization and destabilization of the 5*f* and 6*d* orbitals as *Z* is increased. In this study a demarcation between the chemistry of Pa and U was demonstrated, where the increased participation of the 5*f* electrons and orbitals drives a change in chemistry from that typically encountered for the *d*-transition metals to that typified by the chemistry of the 5*f* actinide elements U, Np and Pu. Using density functional theory and analysis of the bonding in these clusters using the QTAIM approach, we suggest that the increasing dominance of the 5*f* orbitals and their participation in the chemical bonding is responsible for a change in molecular structure not necessarily caused by increased spatial overlap of the actinide and ligand orbitals, but rather closer matching of the actinide and ligand orbital energies in these systems. Such periodic trends in the electronic structure and properties of the early and middle actinide have been shown to have significant impact on the chemistry of the actinide elements particularly with respect to the stabilization of exotic oxidation states, the formation of metal–ligand multiple bonds, and the overall chemistry of these economically important elements.

## Methods

**Caution!** ^231^Pa (*t*_1/2_ = 33,000 years) is an alpha-emitting radionuclide, and along with its decay daughters poses significant radiological hazards. All work was performed using strict radiological controls in a facility designated and designed for the use of alpha-emitting radionuclides.

### Synthesis of [Pa_4_O(O_2_)_6_F_12_]^6−^

The fluoroprotactinate salts of Rb_2_PaF_7_, Cs_2_PaF_7_, and ((CH_3_)_4_N)PaF_6_ were synthesized as described previously and used as precursors to the reported compounds^39^. The total ^231^Pa used for each reaction was 5 mg, 0.022 mmol. The precursors were crystallized in small PTFE cups under ambient conditions. Aliquots of 30% wt H_2_O_2(aq)_ (200 µL, 1.9 mmol) were added to the dry crystals, and the crystals readily dissolved. Evaporation of this solution resulted in the formation of a wet crystalline mass from which irregular fragments of the reported compounds could be harvested for single crystal X-ray diffraction studies.

### Crystallographic studies

Single crystal X-ray diffraction data were collected using a Bruker APEX II X-ray diffractometer. The diffractometer is equipped with a graphite monochromated X-ray source using Mo *K*_α_ radiation (*λ* = 0.71073 Å). Reflection intensities were collected using four orientations of the crystal in φ, and 0.5° scans in ω over 180°. All the data were collected at 100 K using and Oxford Cryosystems 700 series cryostat. The unit cells were determined using the APEX II suite of software. Corrections for absorption were performed using SADABS^40^. Structure solutions and refinement were carried out using SHELXS and SHELXL respectively^41^.

### Computational details

All cluster geometries were optimized with the Turbomole package at the density functional theory level (DFT), using the PBE0 density functional with an ultrafine integration grid, m4, and converging the structure when the Cartesian norm reached 10^−4^ atomic units^42,43^. The PBE0 functional was chosen as it gives accurate structural parameters for actinide species^44^. The minima were characterized by harmonic vibrational analysis. Small-core relativistic effective core potentials were employed for Nb, Ta, and Pa, Rb, Cs, to replace 28 core electrons of Nb, Rb, 46 core electron of Cs, and 60 of Ta, Pa and U^45–49^. The valence electrons are described with triple-zeta quality basis sets of dhf-TZVPP type for Nb, and Ta and the def2-TZVPP basis sets that optimized by Cao and Dolg for U and Pa^45,46^. The fluorine and oxygen atoms are described by def2-TZVPP basis sets^50^. Single-point energy calculations were performed in a water solvent modeled as a dielectric continuum (COSMO) with a dielectric constant of 80.1^51^. These are used to compute the relative formation energies of reaction (1) reported in Table 3, which are reported as the difference with respect to niobium to discuss trends that should be minorly affected by unbalanced errors inherent to the electronic structure parameters (functional and basis sets) and the COSMO solvent model^52^. Metal–ligand chemical bonds were analyzed through the topology of the electronic density with the QTAIM theory implemented in the AIMall package^34,53,54^. Natural Localized Molecular Orbitals (NLMOs) were computed with the NBO6 program^55^.

### Data availability

The data that support the findings of this study are available from the corresponding authors upon reasonable request. Crystallographic information files have been depostited with the Cambridge Crystal Structure Database under accession codes 1588065 (Rb), 1588066 (Cs), 1588067 (Me_4_N). These data can be obtained free of charge from The Cambridge Crystallographic Data Centre via the Internet at www.ccdc.cam.ac.uk/data_request/cif. The data supporting the quantum chemical calculations and structures are available on the free platform Zenodo with the identifier 10.5281/zenodo.1134640.

## Electronic supplementary material

Supplementary Information
