# Supplementary material for: Protactinium and the intersection of actinide and transition metal chemistry
Source: Nat Commun. 2018 Feb 12;9:622. doi: 10.1038/s41467-018-02972-z (PMC5809381; doi:10.1038/s41467-018-02972-z)
Supplement: Supplementary file 1 — Supplementary Information [file 41467_2018_2972_MOESM1_ESM.pdf]

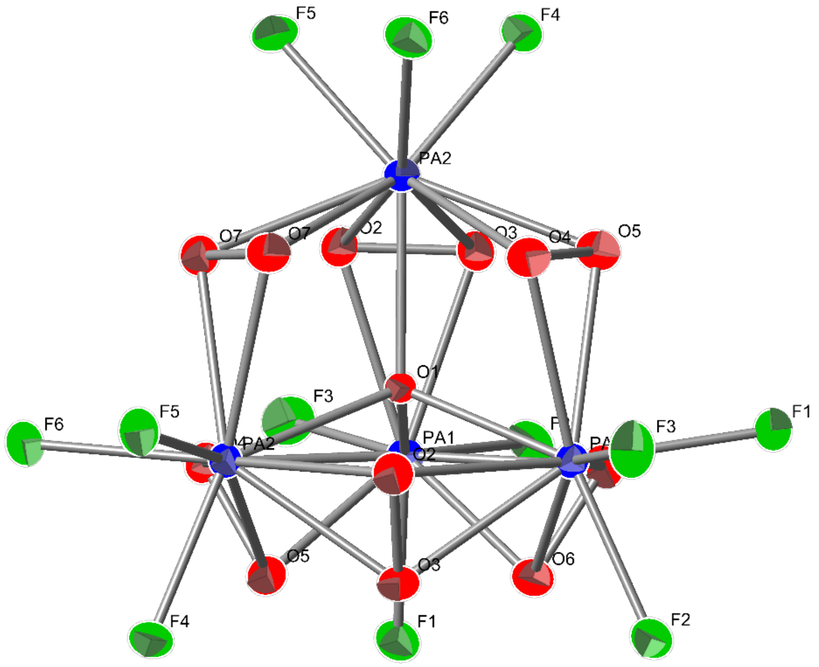

Protactinium in blue, Fluorine in green, Oxygen in red

Supplementary Figure 1. ORTEP Plot of $Rb_6[Pa_4O(O_2)_6F_{12}]\cdot(H_2O_2)_4$ [1]. Thermal ellipsoids at 50% showing only the tetranuclear Pa cluster for clarity.

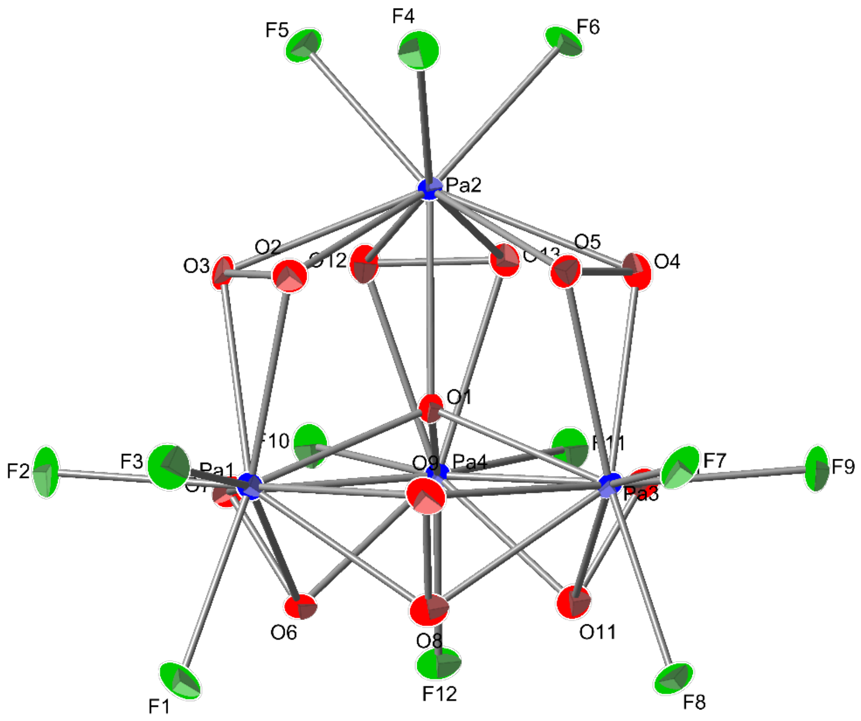

Protactinium in blue, Fluorine in green, Oxygen in red

Supplementary Figure 2. ORTEP Plot of $(Me_4N)_7[Pa_4O(O_2)_6F_{12}]\cdot(H_2O)_{15}F$ [2] Ellipsoids at 50% showing only the tetranuclear Pa cluster for clarity

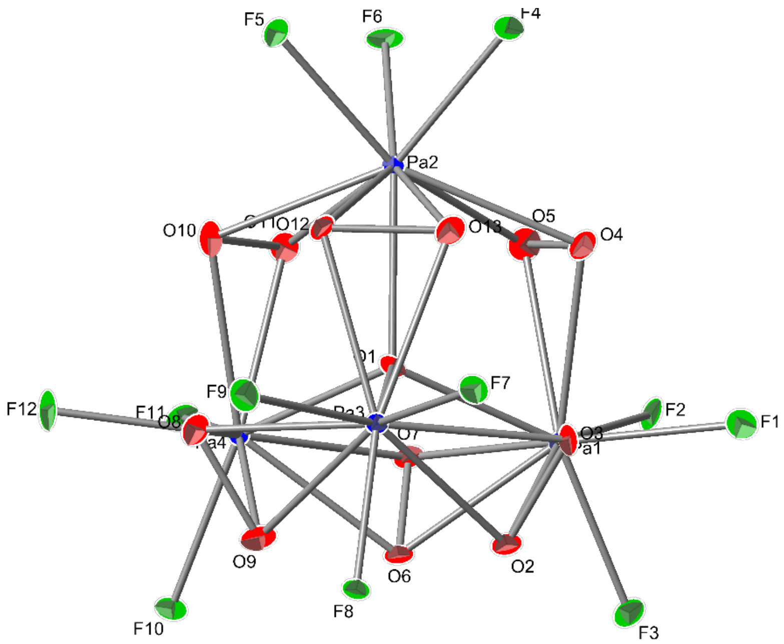

Protactinium in blue, Fluorine in green, Oxygen in red

Supplementary Figure 3. ORTEP Plot of $Cs_6[Pa_4O(O_2)_6F_{12}]\cdot(H_2O)_{10}$ [3]. Thermal ellipsoids at 50% showing only the tetranuclear Pa cluster for clarity.

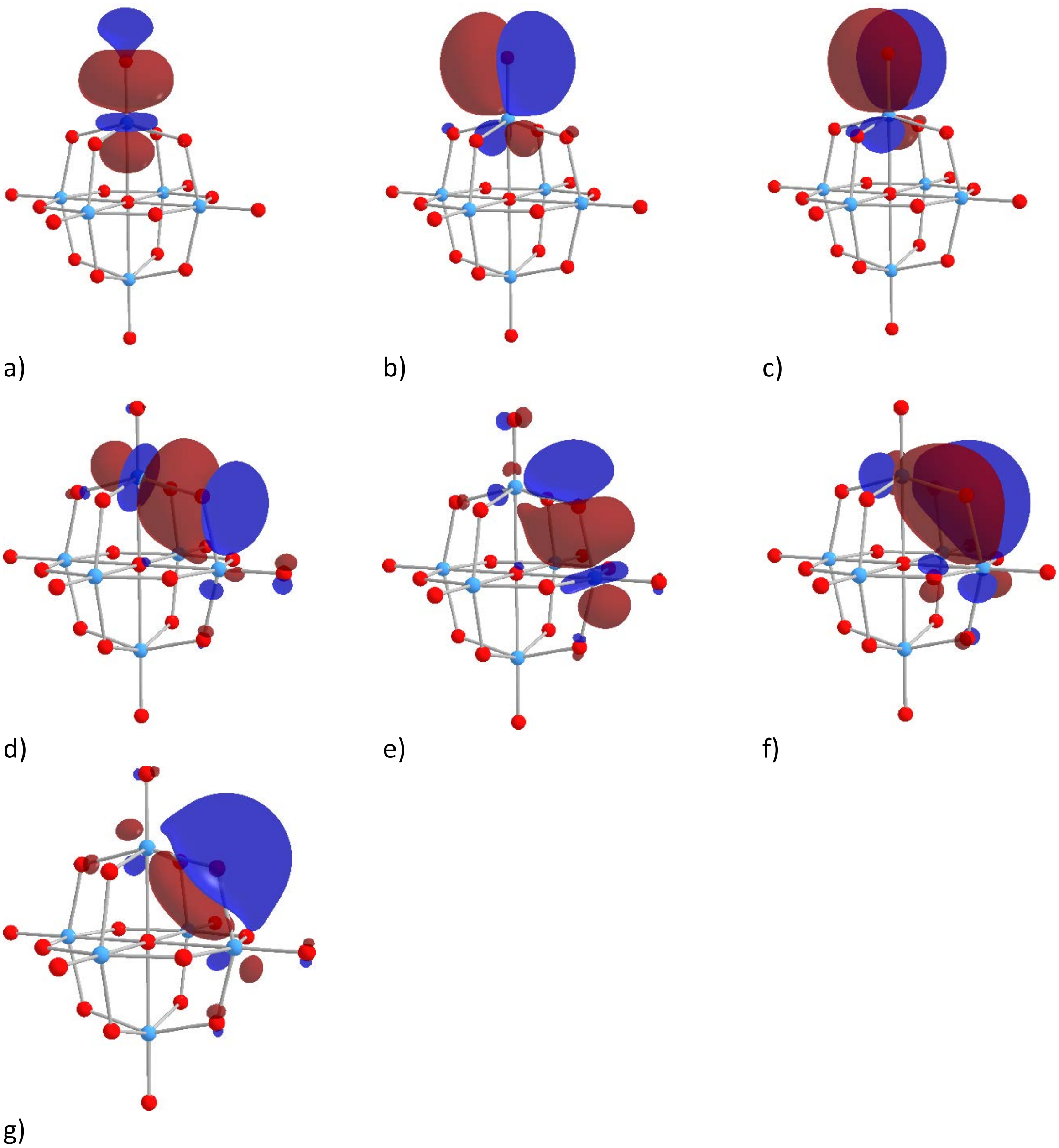

Supplementary Figure 4. Plots of the natural localized molecular orbitals for $Ta_6O_{19}^{6-}$. Panel (a) showing the σ and two π (b, c) bonds in the Ta-O_{yl} interaction, and the two σ bonds (d, e) plus the 3 center 2 electron π bonds (f, g) in the Ta-μ_2O vertices of the $[Ta_6O_{19}]^{8-}$ cluster. The isosurface cut-off is 0.03.

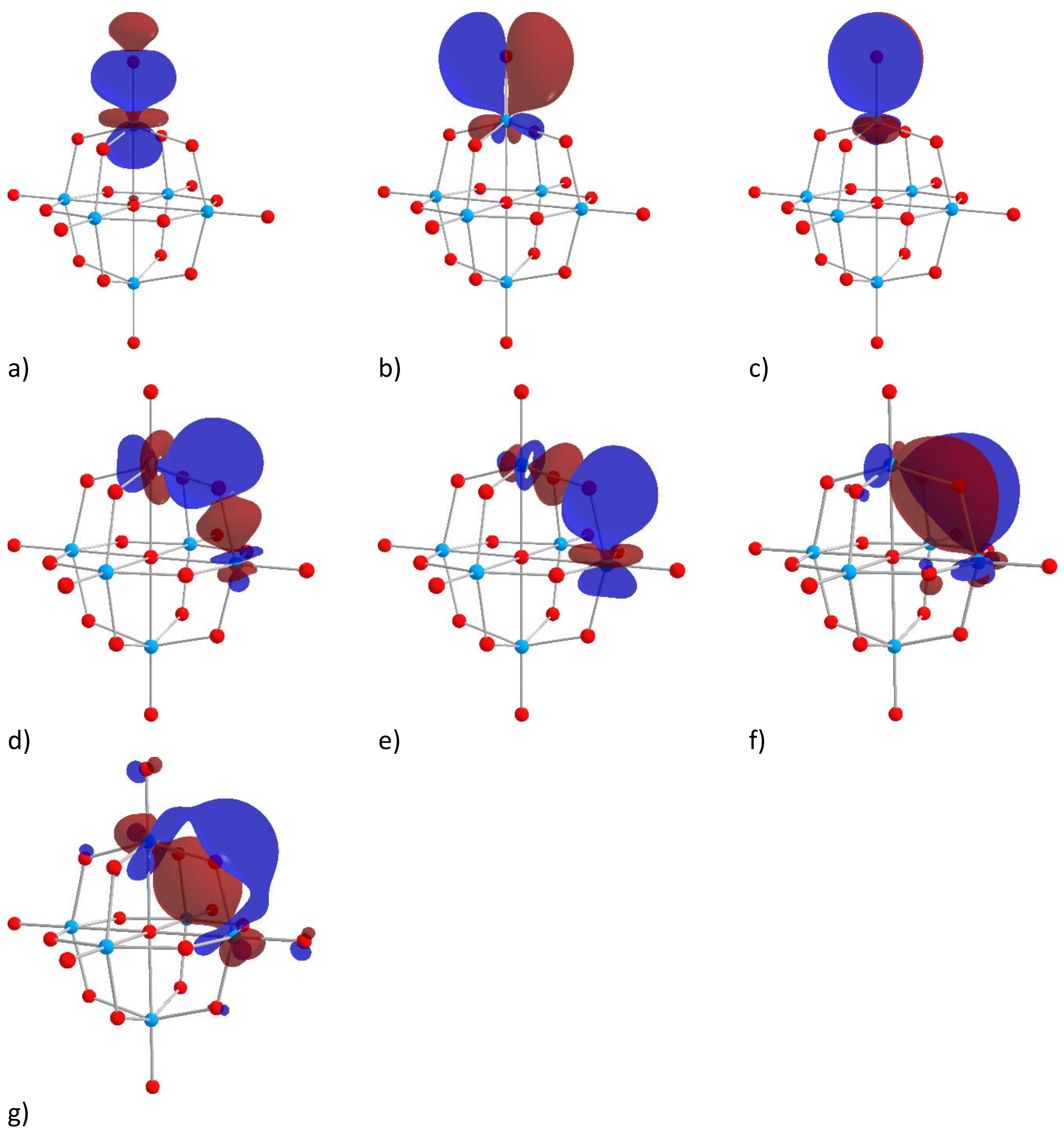

Supplementary Figure 5. Plots of the natural localized molecular orbitals for $Pa_6O_{19}^{6-}$. Showing the σ (a) and two π (b, c) bonds in the Pa-O_{yl} interaction, and the two, and the two σ bonds (d, e), plus the 3 centers 2 electrons π bonds (f, g) in the Pa-μ_2O vertices of the $[Pa_6O_{19}]^{8-}$ cluster. The isosurface cut-off is 0.03.

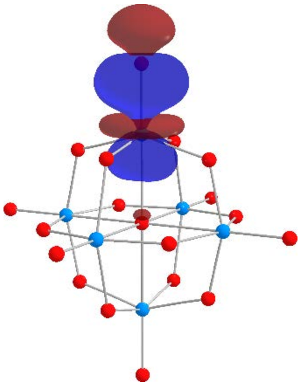

a)

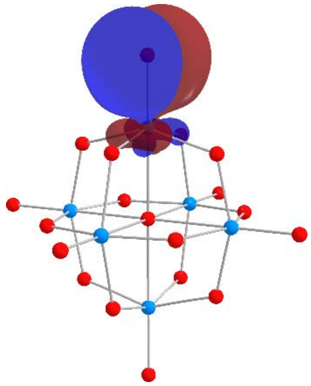

b)

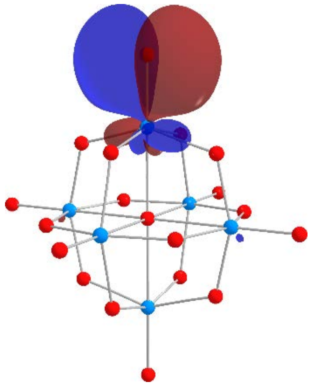

c)

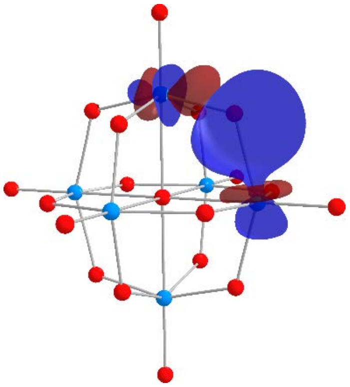

d)

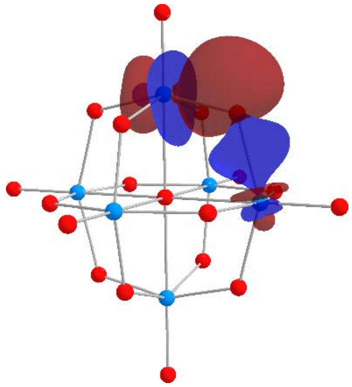

e)

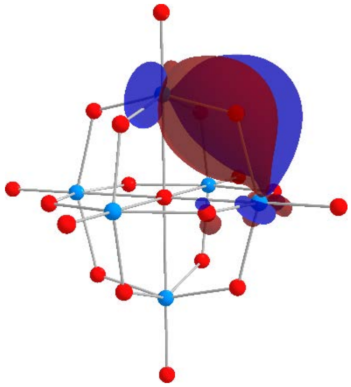

f)

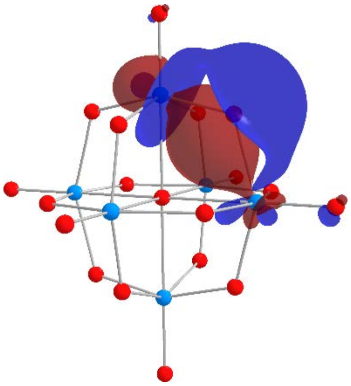

g)

Supplementary Figure 6. Plots of the natural localized molecular orbitals for $U_6O_{19}^{6-}$. Showing the σ (a) and two π (b, c) bonds in the U-O_{yl} interaction, the σ bond (d) to the longest U- μ_2O oxygen, the σ bond (e) to the U- μ_2O shortest and two π (f, g) bonds essentially the shortest U- μ_2O interaction in the $[U_6O_{19}]^{8-}$ cluster. The isosurface cut-off is 0.03.

Supplementary Table 1. Crystallographic Parameters $Rb_6[Pa_4O(O_2)_6F_{12}]\cdot(H_2O_2)_4$ [1]

**Formula**	**$Rb_6[Pa_4O(O_2)_6F_{12}]\cdot(H_2O_2)_4$**
Formula Weight (g mol^{-1})	2000.98
Crystal System	Monoclinic
Space Group	*C* 2/c (No. 15)
a, b, c (Å)	18.030(1), 10.750(1), 15.665(1)
α, β, γ (deg.)	90, 110.650(1), 90
Volume ($Å^3$)	2840.8(4)
Z	4
ρ (g cm^{-3})	4.679
μ [Mo(K_α)] (mm^{-1})	32.202
F(000)	3448
Crystal Size (mm)	0.200 0.100 0.100
T (K)	100
R, wR^2, S	0.0268, 0.0698, 1.04

Supplementary Table 2. Cystallographic Parameters for $(Me_4N)_7[Pa_4O(O_2)_6F_{12}]\cdot(H_2O)_{15}F$ [2]

**Formula**	**$(Me_4N)_7[Pa_4O(O_2)_6F_{12}]\cdot(H_2O)_{15}F$**
Formula Weight (g mol^{-1})	2135.02
Crystal System	Monoclinic
Space Group	$P\ 2_1/c$ (No. 14)
a, b, c (Å)	21.021(1), 16.433(1), 20.631(1)
α, β, γ（deg.)	90, 110.887(1), 90
Volume ($Å^3$)	6658.6(6)
Z	4
ρ (g cm^{-3})	2.130
μ [$Mo(K_α)$] (mm^{-1})	9.424
F(000)	4020
Crystal Size (mm)	0.150 0.100 0.100
T (K)	100
R, wR^2, S	0.053, 0.107, 1.04

Supplementary Table 3. Cystallographic Parameters for $Cs_6[Pa_4O(O_2)_6F_{12}]\cdot(H_2O)_{10}$ [3]

Formula	$Cs_6[Pa_4O(O_2)_6F_{12}]\cdot(H_2O)_{10}$
Formula Weight (g mol^{-1})	2135.02
Crystal System	Monoclinic
Space Group	*P* 2_1/c (No. 14)
a, b, c (Å)	15.787(1), 10.025(1), 20.631(2)
α, β, γ (deg.)	90, 93.75(2), 90
Volume (Å^3)	3257.9(5)
Z	4
ρ (g cm^{-3})	4.725
μ [Mo(K_α)] (mm^{-1})	25.789
F(000)	3944
Crystal Size (mm)	0.150 0.100 0.100
T (K)	100
R, wR^2, S	0.038, 0.078, 1.03

Supplementary Table 4. Selected Bond Distances and Angles for [1]

Bond Distances (Angstroms)

Pa1	-F1	2.232(3)	Pa2	-O2	2.329(4)
Pa1	-F2	2.157(3)	Pa2	-O3	2.383(4)
Pa1	-F3	2.161(4)	Pa2	-O7	2.347(4)
Pa1	-O1	2.312(3)	Pa2	-O4_a	2.373(4)
Pa1	-O2	2.386(4)	Pa2	-O5_a	2.338(4)
Pa1	-O3	2.363(4)	Pa2	-O7_a	2.394(4)
Pa1	-O4	2.362(4)	Pa2	-F4_b	2.167(3)
Pa1	-O5	2.393(4)	O2	-O3	1.486(6)
Pa1	-O6	2.343(4)	O4	-O5	1.477(6)
Pa1	-O6_a	2.380(4)	O6	-O6_a	1.484(6)
Pa2	-F5	2.158(3)	O7	-O7_a	1.485(6)
Pa2	-F6	2.248(4)	O8	-O9	1.446(11)
Pa2	-O1	2.316(3)	O10	-O11	1.468(9)

Bond Angles (Degrees)

F1	-Pa1	-F2	76.15(12)	O1	-Pa1	-O6	69.93(15)
F1	-Pa1	-F3	78.71(14)	O1	-Pa1	-O6_a	69.29(14)
F1	-Pa1	-O1	133.33(9)	O2	-Pa1	-O3	36.48(14)
F1	-Pa1	-O2	151.29(13)	O2	-Pa1	-O4	74.52(15)
F1	-Pa1	-O3	153.29(13)	O2	-Pa1	-O5	107.54(15)
F1	-Pa1	-O4	95.09(13)	O2	-Pa1	-O6	133.43(14)
F1	-Pa1	-O5	73.09(13)	O2	-Pa1	-O6_a	107.24(14)
F1	-Pa1	-O6	75.13(13)	O3	-Pa1	-O4	108.26(15)
F1	-Pa1	-O6_a	99.25(14)	O3	-Pa1	-O5	133.62(15)
F2	-Pa1	-F3	75.92(15)	O3	-Pa1	-O6	107.99(14)
F2	-Pa1	-O1	136.13(12)	O3	-Pa1	-O6_a	74.04(14)
F2	-Pa1	-O2	99.97(13)	O4	-Pa1	-O5	36.19(15)
F2	-Pa1	-O3	77.14(14)	O4	-Pa1	-O6	108.88(15)

F2	-Pa1	-O4	151.24(14)	O4	-Pa1	-O6_a	133.85(14)
F2	-Pa1	-O5	149.22(13)	O5	-Pa1	-O6	75.58(15)
F2	-Pa1	-O6	95.46(14)	O5	-Pa1	-O6_a	108.75(14)
F2	-Pa1	-O6_a	74.90(14)	O6	-Pa1	-O6_a	36.61(15)
F3	-Pa1	-O1	132.85(16)	F5	-Pa2	-F6	75.45(14)
F3	-Pa1	-O2	72.82(15)	F5	-Pa2	-O1	135.15(12)
F3	-Pa1	-O3	94.41(15)	F5	-Pa2	-O2	78.79(14)
F3	-Pa1	-O4	75.50(15)	F5	-Pa2	-O3	102.88(14)
F3	-Pa1	-O5	99.02(15)	F5	-Pa2	-O7	93.19(13)
F3	-Pa1	-O6	153.74(15)	F5	-Pa2	-O4_a	147.39(14)
F3	-Pa1	-O6_a	150.34(16)	F5	-Pa2	-O5_a	153.04(14)
O1	-Pa1	-O2	68.55(13)	F5	-Pa2	-O7_a	73.34(13)
O1	-Pa1	-O3	69.40(10)	F4_b	-Pa2	-F5	77.14(12)
O1	-Pa1	-O4	69.03(13)	F6	-Pa2	-O1	132.97(9)
O1	-Pa1	-O5	69.00(10)	F6	-Pa2	-O2	154.12(14)
F6	-Pa2	-O3	150.30(13)	O4_a	-Pa2	-O5_a	36.53(15)
F6	-Pa2	-O7	75.30(13)	O4_a	-Pa2	-O7_a	106.96(15)
F6	-Pa2	-O4_a	72.36(14)	F4_b	-Pa2	-O4_a	99.64(14)
F6	-Pa2	-O5_a	93.50(14)	O5_a	-Pa2	-O7_a	133.37(15)
F6	-Pa2	-O7_a	99.60(14)	F4_b	-Pa2	-O5_a	76.32(14)
F4_b	-Pa2	-F6	75.85(12)	F4_b	-Pa2	-O7_a	150.29(14)
O1	-Pa2	-O2	69.45(11)	Pa1	-O1	-Pa2	109.51(1)
O1	-Pa2	-O3	68.97(13)	Pa1	-O1	-Pa1_a	109.0(2)
O1	-Pa2	-O7	69.36(15)	Pa1	-O1	-Pa2_a	109.52(1)
O1	-Pa2	-O4_a	68.76(10)	Pa1_a	-O1	-Pa2	109.52(1)
O1	-Pa2	-O5_a	69.88(13)	Pa2	-O1	-Pa2_a	109.8(2)
O1	-Pa2	-O7_a	68.54(15)	Pa1_a	-O1	-Pa2_a	109.51(1)
F4_b	-Pa2	-O1	135.56(14)	Pa1	-O2	-Pa2	106.58(16)
O2	-Pa2	-O3	36.76(14)	Pa1	-O2	-O3	70.9(2)
O2	-Pa2	-O7	108.90(14)	Pa2	-O2	-O3	73.6(2)

O2	-Pa2	-O4_a	133.53(15)	Pa1	-O3	-Pa2	105.60(17)
O2	-Pa2	-O5_a	108.64(15)	Pa1	-O3	-O2	72.6(2)
O2	-Pa2	-O7_a	75.12(14)	Pa2	-O3	-O2	69.7(2)
F4_b	-Pa2	-O2	96.06(14)	Pa1	-O4	-O5	73.0(2)
O3	-Pa2	-O7	134.04(14)	Pa1	-O4	-Pa2_a	105.95(16)
O3	-Pa2	-O4_a	107.58(14)	Pa2_a	-O4	-O5	70.4(2)
O3	-Pa2	-O5_a	74.58(14)	Pa1	-O5	-O4	70.8(2)
O3	-Pa2	-O7_a	108.39(14)	Pa1	-O5	-Pa2_a	106.08(16)
F4_b	-Pa2	-O3	74.92(14)	Pa2_a	-O5	-O4	73.0(2)
O4_a	-Pa2	-O7	73.87(15)	Pa1	-O6	-Pa1_a	105.63(17)
O5_a	-Pa2	-O7	107.92(15)	Pa1	-O6	-O6_a	73.1(2)
O7	-Pa2	-O7_a	36.49(14)	Pa1_a	-O6	-O6_a	70.3(2)
F4_b	-Pa2	-O7	151.05(14)	Pa2	-O7	-Pa2_a	106.16(16)
Pa2	-O7	-O7_a	73.5(2)	Pa2_a	-O7	-O7_a	70.0(2)

Translation of Symmetry Code to Equiv.Pos

a = -x,y,1/2-z   b = -1/2+x,1/2-y,-1/2+z

Supplementary Table 5. Selected Bond Distances and Angles for [2]

Pa1	-F1	2.230(6)	Pa3	-O10	2.351(7)
Pa1	-F2	2.192(6)	Pa3	-O11	2.365(7)
Pa1	-F3	2.189(6)	Pa4	-F10	2.210(5)
Pa1	-O1	2.310(6)	Pa4	-F11	2.158(6)
Pa1	-O2	2.349(7)	Pa4	-F12	2.206(5)
Pa1	-O3	2.350(7)	Pa4	-O1	2.305(6)
Pa1	-O6	2.348(7)	Pa4	-O6	2.384(6)
Pa1	-O7	2.378(6)	Pa4	-O7	2.357(6)
Pa1	-O8	2.361(6)	Pa4	-O10	2.376(6)
Pa1	-O9	2.355(6)	Pa4	-O11	2.368(6)
Pa2	-F4	2.201(7)	Pa4	-O12	2.361(7)
Pa2	-F5	2.164(6)	Pa4	-O13	2.380(7)

Pa2	-F6	2.195(5)	O2	-O3	1.486(9)
Pa2	-O1	2.303(6)	O4	-O5	1.487(9)
Pa2	-O2	2.368(6)	O6	-O7	1.491(9)
Pa2	-O3	2.372(6)	O8	-O9	1.491(9)
Pa2	-O4	2.379(6)	O10	-O11	1.480(9)
Pa2	-O5	2.355(6)	O12	-O13	1.481(8)
Pa2	-O12	2.361(7)	N1	-C1	1.484(13)
Pa2	-O13	2.344(7)	N1	-C2	1.485(13)
Pa3	-F7	2.204(6)	N1	-C3	1.498(13)
Pa3	-F8	2.188(6)	N1	-C4	1.508(14)
Pa3	-F9	2.198(4)	C1	-H1A	0.9800
Pa3	-O1	2.336(6)	C1	-H1B	0.9800
Pa3	-O4	2.355(7)	C1	-H1C	0.9800
Pa3	-O5	2.373(7)	C2	-H2A	0.9800
Pa3	-O8	2.363(6)	C2	-H2B	0.9800
Pa3	-O9	2.359(6)	C2	-H2C	0.9800
N2	-C6	1.514(14)	N4	-C13	1.507(15)
N2	-C7	1.477(13)	N4	-C14	1.480(13)
N2	-C8	1.476(13)	C9	-H9B	0.9800
N2	-C5	1.508(14)	C9	-H9C	0.9800
C3	-H3A	0.9800	C9	-H9A	0.9800
C3	-H3B	0.9800	C10	-H10A	0.9800
C3	-H3C	0.9800	C10	-H10B	0.9800
C4	-H4B	0.9800	C10	-H10C	0.9800
C4	-H4C	0.9800	C11	-H11A	0.9800
C4	-H4A	0.9800	C11	-H11B	0.9800
N3	-C12	1.485(14)	C11	-H11C	0.9800
N3	-C11	1.460(13)	C12	-H12B	0.9800
N3	-C9	1.499(14)	C12	-H12C	0.9800

N3	-C10	1.501(14)	C12	-H12A	0.9800
C5	-H5B	0.9800	N5	-C20	1.531(16)
C5	-H5C	0.9800	N5	-C19	1.486(16)
C5	-H5A	0.9800	N5	-C17	1.473(14)
C6	-H6B	0.9800	N5	-C18	1.469(16)
C6	-H6C	0.9800	C13	-H13B	0.9800
C6	-H6D	0.9800	C13	-H13C	0.9800
C7	-H7A	0.9800	C13	-H13A	0.9800
C7	-H7B	0.9800	C14	-H14A	0.9800
C7	-H7C	0.9800	C14	-H14B	0.9800
C8	-H8B	0.9800	C14	-H14C	0.9800
C8	-H8C	0.9800	C15	-H15A	0.9800
C8	-H8A	0.9800	C15	-H15B	0.9800
N4	-C16	1.517(14)	C15	-H15C	0.9800
N4	-C15	1.482(16)	C16	-H16B	0.9800
C16	-H16C	0.9800	C21	-H21A	0.9800
C16	-H16A	0.9800	C22	-H22A	0.9800
N6	-C24	1.496(15)	C22	-H22B	0.9800
N6	-C23	1.483(17)	C22	-H22C	0.9800
N6	-C21	1.487(13)	C23	-H23A	0.9800
N6	-C22	1.500(17)	C23	-H23B	0.9800
C17	-H17B	0.9800	C23	-H23C	0.9800
C17	-H17C	0.9800	C24	-H24B	0.9800
C17	-H17A	0.9800	C24	-H24C	0.9800
C18	-H18A	0.9800	C24	-H24A	0.9800
C18	-H18B	0.9800	O20	-O29	1.313(19)
C18	-H18C	0.9800	C25	-H25A	0.9800
C19	-H19A	0.9800	C25	-H25B	0.9800
C19	-H19B	0.9800	C25	-H25C	0.9800

C19	-H19C	0.9800	C26	-H26A	0.9800
C20	-H20B	0.9800	C26	-H26B	0.9800
C20	-H20C	0.9800	C26	-H26C	0.9800
C20	-H20A	0.9800	C27	-H27B	0.9800
N7	-C28	1.45(2)	C27	-H27C	0.9800
N7	-C27	1.498(18)	C27	-H27A	0.9800
N7	-C25	1.478(16)	C28	-H28B	0.9800
N7	-C26	1.454(17)	C28	-H28C	0.9800
C21	-H21B	0.9800	C28	-H28A	0.9800
C21	-H21C	0.9800			

Bond Angles (degrees)

F1	-Pa1	-F2	77.3(2)	O1	-Pa1	-O8	69.3(2)
F1	-Pa1	-F3	76.0(2)	O1	-Pa1	-O9	68.8(2)
F1	-Pa1	-O1	134.0(2)	O2	-Pa1	-O3	36.9(2)
F1	-Pa1	-O2	149.6(2)	O2	-Pa1	-O6	134.3(2)
F1	-Pa1	-O3	153.6(2)	O2	-Pa1	-O7	108.9(2)
F1	-Pa1	-O6	75.8(2)	O2	-Pa1	-O8	108.0(2)
F1	-Pa1	-O7	99.3(2)	O2	-Pa1	-O9	74.0(2)
F1	-Pa1	-O8	72.7(2)	O3	-Pa1	-O6	108.7(2)
F1	-Pa1	-O9	95.1(2)	O3	-Pa1	-O7	75.1(2)
F2	-Pa1	-F3	77.5(2)	O3	-Pa1	-O8	133.7(2)
F2	-Pa1	-O1	134.34(18)	O3	-Pa1	-O9	107.7(2)
F2	-Pa1	-O2	99.5(2)	O6	-Pa1	-O7	36.8(2)
F2	-Pa1	-O3	76.3(2)	O6	-Pa1	-O8	73.8(2)
F2	-Pa1	-O6	95.8(2)	O6	-Pa1	-O9	107.6(2)
F2	-Pa1	-O7	73.8(2)	O7	-Pa1	-O8	107.6(2)
F2	-Pa1	-O8	149.8(2)	O7	-Pa1	-O9	133.3(2)
F2	-Pa1	-O9	152.9(2)	O8	-Pa1	-O9	36.9(2)
F3	-Pa1	-O1	133.9(2)	F4	-Pa2	-F5	77.4(2)

F3	-Pa1	-O2	73.8(2)	F4	-Pa2	-F6	77.5(2)
F3	-Pa1	-O3	96.4(2)	F4	-Pa2	-O1	133.4(2)
F3	-Pa1	-O6	151.8(2)	F4	-Pa2	-O2	73.5(2)
F3	-Pa1	-O7	151.2(2)	F4	-Pa2	-O3	95.9(2)
F3	-Pa1	-O8	98.1(2)	F4	-Pa2	-O4	99.9(2)
F3	-Pa1	-O9	75.4(2)	F4	-Pa2	-O5	75.4(2)
O1	-Pa1	-O2	69.1(2)	F4	-Pa2	-O12	151.0(2)
O1	-Pa1	-O3	69.0(2)	F4	-Pa2	-O13	152.9(2)
O1	-Pa1	-O6	69.5(2)	F5	-Pa2	-F6	78.1(2)
O1	-Pa1	-O7	69.4(2)	F5	-Pa2	-O1	133.4(2)
F5	-Pa2	-O2	97.9(2)	O4	-Pa2	-O5	36.6(2)
F5	-Pa2	-O3	75.0(2)	O4	-Pa2	-O12	106.7(2)
F5	-Pa2	-O4	151.3(2)	O4	-Pa2	-O13	73.4(2)
F5	-Pa2	-O5	152.8(2)	O5	-Pa2	-O12	133.4(2)
F5	-Pa2	-O12	73.8(2)	O5	-Pa2	-O13	107.6(2)
F5	-Pa2	-O13	95.9(2)	O12	-Pa2	-O13	36.7(2)
F6	-Pa2	-O1	134.0(2)	F7	-Pa3	-F8	78.1(2)
F6	-Pa2	-O2	150.8(2)	F7	-Pa3	-F9	76.3(2)
F6	-Pa2	-O3	153.1(2)	F7	-Pa3	-O1	133.9(2)
F6	-Pa2	-O4	73.5(2)	F7	-Pa3	-O4	98.4(2)
F6	-Pa2	-O5	94.4(2)	F7	-Pa3	-O5	75.2(2)
F6	-Pa2	-O12	99.2(2)	F7	-Pa3	-O8	97.7(2)
F6	-Pa2	-O13	75.5(2)	F7	-Pa3	-O9	75.3(2)
O1	-Pa2	-O2	68.9(2)	F7	-Pa3	-O10	151.3(2)
O1	-Pa2	-O3	68.7(2)	F7	-Pa3	-O11	152.1(2)
O1	-Pa2	-O4	68.6(2)	F8	-Pa3	-F9	77.7(2)
O1	-Pa2	-O5	69.7(2)	F8	-Pa3	-O1	131.6(2)
O1	-Pa2	-O12	68.9(2)	F8	-Pa3	-O4	154.5(2)
O1	-Pa2	-O13	69.5(2)	F8	-Pa3	-O5	153.1(2)

O2	-Pa2	-O3	36.5(2)	F8	-Pa3	-O8	72.0(2)
O2	-Pa2	-O4	108.8(2)	F8	-Pa3	-O9	95.6(2)
O2	-Pa2	-O5	76.0(2)	F8	-Pa3	-O10	98.6(2)
O2	-Pa2	-O12	107.5(2)	F8	-Pa3	-O11	74.0(2)
O2	-Pa2	-O13	133.6(2)	F9	-Pa3	-O1	135.90(18)
O3	-Pa2	-O4	133.4(2)	F9	-Pa3	-O4	76.9(2)
O3	-Pa2	-O5	109.4(2)	F9	-Pa3	-O5	98.5(2)
O3	-Pa2	-O12	73.9(2)	F9	-Pa3	-O8	149.7(2)
O3	-Pa2	-O13	107.8(2)	F9	-Pa3	-O9	151.6(2)
F9	-Pa3	-O10	75.2(2)	F10	-Pa4	-O10	151.8(2)
F9	-Pa3	-O11	96.1(2)	F10	-Pa4	-O11	152.5(2)
O1	-Pa3	-O4	68.5(2)	F10	-Pa4	-O12	73.7(2)
O1	-Pa3	-O5	68.9(2)	F10	-Pa4	-O13	96.3(2)
O1	-Pa3	-O8	68.9(2)	F11	-Pa4	-F12	77.5(2)
O1	-Pa3	-O9	68.3(2)	F11	-Pa4	-O1	133.9(2)
O1	-Pa3	-O10	69.0(2)	F11	-Pa4	-O6	151.5(2)
O1	-Pa3	-O11	69.4(2)	F11	-Pa4	-O7	151.6(2)
O4	-Pa3	-O5	36.7(2)	F11	-Pa4	-O10	74.4(2)
O4	-Pa3	-O8	133.3(2)	F11	-Pa4	-O11	96.1(2)
O4	-Pa3	-O9	108.0(2)	F11	-Pa4	-O12	97.7(2)
O4	-Pa3	-O10	72.3(2)	F11	-Pa4	-O13	75.1(2)
O4	-Pa3	-O11	106.1(2)	F12	-Pa4	-O1	134.08(19)
O5	-Pa3	-O8	108.7(2)	F12	-Pa4	-O6	74.1(2)
O5	-Pa3	-O9	74.9(2)	F12	-Pa4	-O7	95.5(2)
O5	-Pa3	-O10	106.2(2)	F12	-Pa4	-O10	98.4(2)
O5	-Pa3	-O11	132.7(2)	F12	-Pa4	-O11	74.9(2)
O8	-Pa3	-O9	36.8(2)	F12	-Pa4	-O12	151.2(2)
O8	-Pa3	-O10	108.5(2)	F12	-Pa4	-O13	152.6(2)
O8	-Pa3	-O11	75.3(2)	O1	-Pa4	-O6	68.9(2)

O9	-Pa3	-O10	133.2(2)	O1	-Pa4	-O7	69.8(2)
O9	-Pa3	-O11	108.7(2)	O1	-Pa4	-O10	69.0(2)
O10	-Pa3	-O11	36.6(2)	O1	-Pa4	-O11	69.9(2)
F10	-Pa4	-F11	77.4(2)	O1	-Pa4	-O12	68.9(2)
F10	-Pa4	-F12	77.6(2)	O1	-Pa4	-O13	68.9(2)
F10	-Pa4	-O1	133.2(2)	O6	-Pa4	-O7	36.7(2)
F10	-Pa4	-O6	98.3(2)	O6	-Pa4	-O10	107.6(2)
F10	-Pa4	-O7	74.2(2)	O6	-Pa4	-O11	74.6(2)
O6	-Pa4	-O12	108.3(2)	Pa2	-O5	-O4	72.6(3)
O6	-Pa4	-O13	133.4(2)	Pa3	-O5	-O4	71.0(4)
O7	-Pa4	-O10	134.0(2)	Pa1	-O6	-Pa4	105.6(3)
O7	-Pa4	-O11	108.72(19)	Pa1	-O6	-O7	72.7(4)
O7	-Pa4	-O12	75.1(2)	Pa4	-O6	-O7	70.7(3)
O7	-Pa4	-O13	108.6(2)	Pa1	-O7	-Pa4	105.5(2)
O10	-Pa4	-O11	36.4(2)	Pa1	-O7	-O6	70.5(3)
O10	-Pa4	-O12	107.7(2)	Pa4	-O7	-O6	72.6(3)
O10	-Pa4	-O13	74.4(2)	Pa1	-O8	-Pa3	106.6(3)
O11	-Pa4	-O12	133.8(2)	Pa1	-O8	-O9	71.4(3)
O11	-Pa4	-O13	108.0(2)	Pa3	-O8	-O9	71.5(3)
O12	-Pa4	-O13	36.4(2)	Pa1	-O9	-Pa3	106.9(3)
Pa1	-O1	-Pa2	109.7(3)	Pa1	-O9	-O8	71.8(3)
Pa1	-O1	-Pa3	109.2(2)	Pa3	-O9	-O8	71.7(3)
Pa1	-O1	-Pa4	109.5(2)	Pa3	-O10	-Pa4	106.2(2)

Pa2	-O1	-Pa3	109.5(2)	Pa3	-O10	-O11	72.2(4)
Pa2	-O1	-Pa4	109.8(2)	Pa4	-O10	-O11	71.6(3)
Pa3	-O1	-Pa4	109.1(3)	Pa3	-O11	-Pa4	106.0(3)
Pa1	-O2	-Pa2	106.3(3)	Pa3	-O11	-O10	71.2(4)
Pa1	-O2	-O3	71.6(4)	Pa4	-O11	-O10	72.1(3)

Pa2	-O2	-O3	71.9(3)	Pa2	-O12	-Pa4	106.0(2)
Pa1	-O3	-Pa2	106.1(2)	Pa2	-O12	-O13	71.0(4)
Pa1	-O3	-O2	71.5(4)	Pa4	-O12	-O13	72.5(4)
Pa2	-O3	-O2	71.6(3)	Pa2	-O13	-Pa4	105.9(2)
Pa2	-O4	-Pa3	106.3(2)	Pa2	-O13	-O12	72.3(4)
Pa2	-O4	-O5	70.8(3)	Pa4	-O13	-O12	71.1(4)
Pa3	-O4	-O5	72.4(4)	C1	-N1	-C2	110.9(8)
Pa2	-O5	-Pa3	106.5(3)	C1	-N1	-C3	109.9(7)
C1	-N1	-C4	108.6(7)	H4B	-C4	-H4C	110.00
C2	-N1	-C3	108.7(8)	N1	-C4	-H4A	109.00
C2	-N1	-C4	109.5(8)	N1	-C4	-H4B	109.00
C3	-N1	-C4	109.2(8)	N1	-C4	-H4C	109.00
N1	-C1	-H1A	109.00	H4A	-C4	-H4B	110.00
N1	-C1	-H1B	109.00	H4A	-C4	-H4C	110.00
N1	-C1	-H1C	109.00	C11	-N3	-C12	110.1(9)
H1A	-C1	-H1B	110.00	C9	-N3	-C12	109.2(9)
H1A	-C1	-H1C	110.00	C9	-N3	-C10	108.5(8)
H1B	-C1	-H1C	110.00	C9	-N3	-C11	109.7(9)
N1	-C2	-H2A	109.00	C10	-N3	-C11	110.8(8)
N1	-C2	-H2B	110.00	C10	-N3	-C12	108.6(9)
N1	-C2	-H2C	109.00	N2	-C5	-H5B	110.00
H2A	-C2	-H2B	109.00	N2	-C5	-H5A	109.00
H2A	-C2	-H2C	109.00	H5A	-C5	-H5C	109.00
H2B	-C2	-H2C	110.00	N2	-C5	-H5C	110.00
C6	-N2	-C7	108.0(8)	H5A	-C5	-H5B	109.00
C6	-N2	-C8	108.6(8)	H5B	-C5	-H5C	109.00
C7	-N2	-C8	111.3(7)	N2	-C6	-H6D	109.00
C5	-N2	-C7	109.6(8)	N2	-C6	-H6B	109.00
C5	-N2	-C8	109.5(8)	N2	-C6	-H6C	110.00

C5	-N2	-C6	109.8(7)	H6B	-C6	-H6D	109.00
H3A	-C3	-H3B	109.00	H6C	-C6	-H6D	110.00
H3A	-C3	-H3C	109.00	H6B	-C6	-H6C	109.00
N1	-C3	-H3A	109.00	H7A	-C7	-H7B	109.00
N1	-C3	-H3B	109.00	H7A	-C7	-H7C	109.00
N1	-C3	-H3C	110.00	N2	-C7	-H7A	110.00
H3B	-C3	-H3C	110.00	N2	-C7	-H7B	109.00
N2	-C7	-H7C	110.00	N3	-C11	-H11A	110.00
H7B	-C7	-H7C	109.00	N3	-C11	-H11B	110.00
H8B	-C8	-H8C	109.00	N3	-C11	-H11C	109.00
N2	-C8	-H8A	109.00	H11B	-C11	-H11C	109.00
N2	-C8	-H8B	109.00	H12B	-C12	-H12C	109.00
N2	-C8	-H8C	110.00	N3	-C12	-H12A	109.00
H8A	-C8	-H8B	109.00	N3	-C12	-H12B	109.00
H8A	-C8	-H8C	109.00	N3	-C12	-H12C	109.00
C15	-N4	-C16	110.2(9)	H12A	-C12	-H12B	109.00
C13	-N4	-C16	108.9(9)	H12A	-C12	-H12C	109.00
C13	-N4	-C14	110.0(8)	C19	-N5	-C20	107.9(9)
C13	-N4	-C15	110.7(8)	C17	-N5	-C20	109.1(9)
C14	-N4	-C15	108.1(8)	C17	-N5	-C18	111.5(9)
C14	-N4	-C16	108.9(8)	C17	-N5	-C19	110.0(9)
N3	-C9	-H9B	110.00	C18	-N5	-C19	112.7(10)
N3	-C9	-H9A	110.00	C18	-N5	-C20	105.6(10)
H9A	-C9	-H9C	109.00	N4	-C13	-H13B	109.00
N3	-C9	-H9C	109.00	N4	-C13	-H13A	109.00
H9A	-C9	-H9B	109.00	H13A	-C13	-H13C	110.00
H9B	-C9	-H9C	109.00	N4	-C13	-H13C	109.00
N3	-C10	-H10C	109.00	H13A	-C13	-H13B	110.00
N3	-C10	-H10A	109.00	H13B	-C13	-H13C	110.00

N3	-C10	-H10B	109.00	N4	-C14	-H14C	110.00
H10A	-C10	-H10C	110.00	N4	-C14	-H14A	109.00
H10B	-C10	-H10C	109.00	N4	-C14	-H14B	109.00
H10A	-C10	-H10B	110.00	H14A	-C14	-H14C	110.00
H11A	-C11	-H11B	109.00	H14B	-C14	-H14C	110.00
H11A	-C11	-H11C	109.00	H14A	-C14	-H14B	109.00
H15A	-C15	-H15B	110.00	H18B	-C18	-H18C	110.00
H15A	-C15	-H15C	109.00	H18A	-C18	-H18B	109.00
N4	-C15	-H15A	109.00	H19A	-C19	-H19B	109.00
N4	-C15	-H15B	109.00	H19A	-C19	-H19C	109.00
N4	-C15	-H15C	109.00	N5	-C19	-H19A	109.00
H15B	-C15	-H15C	110.00	N5	-C19	-H19B	109.00
H16B	-C16	-H16C	109.00	N5	-C19	-H19C	110.00
N4	-C16	-H16A	109.00	H19B	-C19	-H19C	109.00
N4	-C16	-H16B	109.00	H20B	-C20	-H20C	109.00
N4	-C16	-H16C	109.00	N5	-C20	-H20A	110.00
H16A	-C16	-H16B	110.00	N5	-C20	-H20B	109.00
H16A	-C16	-H16C	109.00	N5	-C20	-H20C	109.00
C23	-N6	-C24	109.0(10)	H20A	-C20	-H20B	110.00
C21	-N6	-C24	109.8(9)	H20A	-C20	-H20C	109.00
C21	-N6	-C22	111.2(9)	C27	-N7	-C28	110.8(11)
C21	-N6	-C23	109.3(9)	C25	-N7	-C28	108.5(11)
C22	-N6	-C23	109.5(9)	C25	-N7	-C26	109.2(10)
C22	-N6	-C24	107.9(10)	C25	-N7	-C27	109.6(11)
N5	-C17	-H17B	109.00	C26	-N7	-C27	110.6(10)
N5	-C17	-H17A	109.00	C26	-N7	-C28	108.1(12)
H17A	-C17	-H17C	109.00	N6	-C21	-H21B	109.00
N5	-C17	-H17C	109.00	N6	-C21	-H21A	109.00
H17A	-C17	-H17B	109.00	H21A	-C21	-H21C	109.00

H17B	-C17	-H17C	109.00	N6	-C21	-H21C	109.00
N5	-C18	-H18C	109.00	H21A	-C21	-H21B	110.00
N5	-C18	-H18A	109.00	H21B	-C21	-H21C	109.00
N5	-C18	-H18B	109.00	N6	-C22	-H22C	109.00
H18A	-C18	-H18C	109.00	N6	-C22	-H22A	109.00
N6	-C22	-H22B	109.00	H25A	-C25	-H25C	110.00
H22A	-C22	-H22C	110.00	H25B	-C25	-H25C	110.00
H22B	-C22	-H22C	109.00	N7	-C26	-H26A	109.00
H22A	-C22	-H22B	109.00	N7	-C26	-H26B	109.00
H23A	-C23	-H23B	110.00	N7	-C26	-H26C	109.00
H23A	-C23	-H23C	110.00	H26A	-C26	-H26B	109.00
N6	-C23	-H23A	109.00	H26A	-C26	-H26C	109.00
N6	-C23	-H23B	110.00	H26B	-C26	-H26C	110.00
N6	-C23	-H23C	109.00	N7	-C27	-H27A	109.00
H23B	-C23	-H23C	109.00	N7	-C27	-H27B	109.00
H24B	-C24	-H24C	110.00	N7	-C27	-H27C	110.00
N6	-C24	-H24A	109.00	H27A	-C27	-H27B	109.00
N6	-C24	-H24B	110.00	H27A	-C27	-H27C	109.00
N6	-C24	-H24C	110.00	H27B	-C27	-H27C	109.00
H24A	-C24	-H24B	109.00	N7	-C28	-H28A	110.00
H24A	-C24	-H24C	109.00	N7	-C28	-H28B	109.00
N7	-C25	-H25A	109.00	N7	-C28	-H28C	110.00
N7	-C25	-H25B	109.00	H28A	-C28	-H28B	109.00
N7	-C25	-H25C	109.00	H28A	-C28	-H28C	109.00
H25A	-C25	-H25B	109.00	H28B	-C28	-H28C	109.00

Supplementary Table 6. Selected Bond Distances and Angles for [3]

Pa1	-F1	2.215(6)	Pa3	-O8	2.389(6)
Pa1	-F2	2.184(5)	Pa3	-O9	2.365(6)

Pa1	-F3	2.177(6)	Pa3	-O12	2.371(6)
Pa1	-O1	2.295(6)	Pa3	-O13	2.410(6)
Pa1	-O2	2.337(6)	Pa4	-F10	2.211(6)
Pa1	-O3	2.371(6)	Pa4	-F11	2.180(5)
Pa1	-O4	2.360(6)	Pa4	-F12	2.242(5)
Pa1	-O5	2.454(7)	Pa4	-O1	2.279(6)
Pa1	-O6	2.361(6)	Pa4	-O6	2.342(6)
Pa1	-O7	2.359(6)	Pa4	-O7	2.359(6)
Pa2	-F4	2.177(6)	Pa4	-O8	2.324(6)
Pa2	-F5	2.208(5)	Pa4	-O9	2.343(6)
Pa2	-F6	2.198(6)	Pa4	-O10	2.369(7)
Pa2	-O1	2.331(6)	Pa4	-O11	2.345(6)
Pa2	-O4	2.386(6)	O2	-O3	1.482(9)
Pa2	-O5	2.328(7)	O4	-O5	1.487(9)
Pa2	-O10	2.349(7)	O6	-O7	1.488(9)
Pa2	-O11	2.346(6)	O8	-O9	1.475(9)
Pa2	-O12	2.373(6)	O10	-O11	1.492(9)
Pa2	-O13	2.335(6)	O12	-O13	1.492(9)
Pa3	-F7	2.164(5)	O14	-O18_a	1.435(11)
Pa3	-F8	2.173(4)	O16	-O20_c	1.462(11)
Pa3	-F9	2.159(6)	O17	-O19_e	1.455(11)
Pa3	-O1	2.333(6)	O21	-O23_g	1.375(13)
Pa3	-O2	2.374(7)	O22	-O22_h	1.481(13)
Pa3	-O3	2.349(6)			

Bond Angles (degrees)

F1	-Pa1	-F2	75.5(2)	O1	-Pa1	-O6	69.2(2)
F1	-Pa1	-F3	76.9(2)	O1	-Pa1	-O7	69.8(2)
F1	-Pa1	-O1	132.1(2)	O2	-Pa1	-O3	36.7(2)
F1	-Pa1	-O2	94.2(2)	O2	-Pa1	-O4	109.4(2)

F1	-Pa1	-O3	71.6(2)	O2	-Pa1	-O5	133.8(2)
F1	-Pa1	-O4	74.7(2)	O2	-Pa1	-O6	74.1(2)
F1	-Pa1	-O5	99.1(2)	O2	-Pa1	-O7	108.4(2)
F1	-Pa1	-O6	151.4(2)	O3	-Pa1	-O4	75.7(2)
F1	-Pa1	-O7	153.9(2)	O3	-Pa1	-O5	108.2(2)
F2	-Pa1	-F3	78.7(2)	O3	-Pa1	-O6	107.6(2)
F2	-Pa1	-O1	133.0(2)	O3	-Pa1	-O7	134.5(2)
F2	-Pa1	-O2	155.1(2)	O4	-Pa1	-O5	35.9(2)
F2	-Pa1	-O3	146.5(2)	O4	-Pa1	-O6	133.6(2)
F2	-Pa1	-O4	90.1(2)	O4	-Pa1	-O7	108.3(2)
F2	-Pa1	-O5	70.9(2)	O5	-Pa1	-O6	107.8(2)
F2	-Pa1	-O6	104.1(2)	O5	-Pa1	-O7	74.8(2)
F2	-Pa1	-O7	78.5(2)	O6	-Pa1	-O7	36.8(2)
F3	-Pa1	-O1	136.6(2)	F4	-Pa2	-F5	76.0(2)
F3	-Pa1	-O2	76.8(2)	F4	-Pa2	-F6	76.1(2)
F3	-Pa1	-O3	99.3(2)	F4	-Pa2	-O1	136.3(2)
F3	-Pa1	-O4	151.3(2)	F4	-Pa2	-O4	75.4(2)
F3	-Pa1	-O5	149.4(2)	F4	-Pa2	-O5	94.9(2)
F3	-Pa1	-O6	75.1(2)	F4	-Pa2	-O10	151.9(2)
F3	-Pa1	-O7	95.4(2)	F4	-Pa2	-O11	147.8(2)
O1	-Pa1	-O2	70.5(2)	F4	-Pa2	-O12	100.0(2)
O1	-Pa1	-O3	70.0(2)	F4	-Pa2	-O13	78.1(2)
O1	-Pa1	-O4	69.0(2)	F5	-Pa2	-F6	76.1(2)
O1	-Pa1	-O5	67.9(2)	F5	-Pa2	-O1	132.6(2)
F5	-Pa2	-O4	150.4(2)	O10	-Pa2	-O11	37.1(2)
F5	-Pa2	-O5	155.0(2)	O10	-Pa2	-O12	76.5(2)
F5	-Pa2	-O10	76.5(2)	O10	-Pa2	-O13	109.8(2)
F5	-Pa2	-O11	101.9(2)	O11	-Pa2	-O12	109.9(2)
F5	-Pa2	-O12	71.5(2)	O11	-Pa2	-O13	133.7(2)

F5	-Pa2	-O13	94.7(2)	O12	-Pa2	-O13	36.9(2)
F6	-Pa2	-O1	135.0(2)	F7	-Pa3	-F8	81.1(2)
F6	-Pa2	-O4	104.0(2)	F7	-Pa3	-F9	76.8(2)
F6	-Pa2	-O5	79.1(2)	F7	-Pa3	-O1	134.7(2)
F6	-Pa2	-O10	91.9(2)	F7	-Pa3	-O2	101.0(2)
F6	-Pa2	-O11	72.3(2)	F7	-Pa3	-O3	77.2(2)
F6	-Pa2	-O12	147.4(2)	F7	-Pa3	-O8	149.3(2)
F6	-Pa2	-O13	154.0(2)	F7	-Pa3	-O9	153.9(2)
O1	-Pa2	-O4	68.0(2)	F7	-Pa3	-O12	93.0(2)
O1	-Pa2	-O5	69.4(2)	F7	-Pa3	-O13	74.1(2)
O1	-Pa2	-O10	69.3(2)	F8	-Pa3	-F9	75.4(2)
O1	-Pa2	-O11	68.4(2)	F8	-Pa3	-O1	133.7(2)
O1	-Pa2	-O12	69.4(2)	F8	-Pa3	-O2	76.7(2)
O1	-Pa2	-O13	68.6(2)	F8	-Pa3	-O3	100.0(2)
O4	-Pa2	-O5	36.7(2)	F8	-Pa3	-O8	95.22(19)
O4	-Pa2	-O10	132.6(2)	F8	-Pa3	-O9	73.0(2)
O4	-Pa2	-O11	106.3(2)	F8	-Pa3	-O12	150.0(2)
O4	-Pa2	-O12	106.2(2)	F8	-Pa3	-O13	155.1(2)
O4	-Pa2	-O13	72.1(2)	F9	-Pa3	-O1	131.8(2)
O5	-Pa2	-O10	107.8(2)	F9	-Pa3	-O2	152.0(2)
O5	-Pa2	-O11	73.3(2)	F9	-Pa3	-O3	154.0(2)
O5	-Pa2	-O12	133.5(2)	F9	-Pa3	-O8	72.8(2)
O5	-Pa2	-O13	106.4(2)	F9	-Pa3	-O9	94.5(2)
F9	-Pa3	-O12	74.6(2)	F10	-Pa4	-O8	98.8(2)
F9	-Pa3	-O13	100.3(2)	F10	-Pa4	-O9	75.6(2)
O1	-Pa3	-O2	69.2(2)	F10	-Pa4	-O10	151.2(2)
O1	-Pa3	-O3	69.7(2)	F10	-Pa4	-O11	150.1(2)
O1	-Pa3	-O8	67.9(2)	F11	-Pa4	-F12	75.0(2)
O1	-Pa3	-O9	68.8(2)	F11	-Pa4	-O1	133.9(2)

O1	-Pa3	-O12	69.4(2)	F11	-Pa4	-O6	99.9(2)
O1	-Pa3	-O13	67.3(2)	F11	-Pa4	-O7	75.9(2)
O2	-Pa3	-O3	36.6(2)	F11	-Pa4	-O8	149.1(2)
O2	-Pa3	-O8	107.9(2)	F11	-Pa4	-O9	152.7(2)
O2	-Pa3	-O9	75.2(2)	F11	-Pa4	-O10	94.7(2)
O2	-Pa3	-O12	133.2(2)	F11	-Pa4	-O11	73.0(2)
O2	-Pa3	-O13	105.9(2)	F12	-Pa4	-O1	134.5(2)
O3	-Pa3	-O8	133.2(2)	F12	-Pa4	-O6	150.4(2)
O3	-Pa3	-O9	108.8(2)	F12	-Pa4	-O7	150.8(2)
O3	-Pa3	-O12	107.3(2)	F12	-Pa4	-O8	74.3(2)
O3	-Pa3	-O13	72.9(2)	F12	-Pa4	-O9	96.6(2)
O8	-Pa3	-O9	36.1(2)	F12	-Pa4	-O10	74.1(2)
O8	-Pa3	-O12	75.1(2)	F12	-Pa4	-O11	97.0(2)
O8	-Pa3	-O13	107.1(2)	O1	-Pa4	-O6	69.8(2)
O9	-Pa3	-O12	108.6(2)	O1	-Pa4	-O7	70.1(2)
O9	-Pa3	-O13	131.9(2)	O1	-Pa4	-O8	69.9(2)
O12	-Pa3	-O13	36.4(2)	O1	-Pa4	-O9	70.1(2)
F10	-Pa4	-F11	77.2(2)	O1	-Pa4	-O10	69.8(2)
F10	-Pa4	-F12	77.1(2)	O1	-Pa4	-O11	69.3(2)
F10	-Pa4	-O1	134.7(2)	O6	-Pa4	-O7	36.9(2)
F10	-Pa4	-O6	73.3(2)	O6	-Pa4	-O8	108.3(2)
F10	-Pa4	-O7	94.9(2)	O6	-Pa4	-O9	74.5(2)
O6	-Pa4	-O10	135.5(2)	Pa1	-O5	-Pa2	105.5(2)
O6	-Pa4	-O11	109.5(2)	Pa1	-O5	-O4	68.6(3)
O7	-Pa4	-O8	135.0(2)	Pa2	-O5	-O4	73.8(3)
O7	-Pa4	-O9	108.7(2)	Pa1	-O6	-Pa4	105.1(2)
O7	-Pa4	-O10	110.1(2)	Pa1	-O6	-O7	71.6(3)
O7	-Pa4	-O11	75.9(2)	Pa4	-O6	-O7	72.2(3)
O8	-Pa4	-O9	36.8(2)	Pa1	-O7	-Pa4	104.7(2)

O8	-Pa4	-O10	74.0(2)	Pa1	-O7	-O6	71.7(3)
O8	-Pa4	-O11	107.8(2)	Pa4	-O7	-O6	70.9(3)
O9	-Pa4	-O10	108.1(2)	Pa3	-O8	-Pa4	106.0(2)
O9	-Pa4	-O11	134.3(2)	Pa3	-O8	-O9	71.0(3)
O10	-Pa4	-O11	36.9(2)	Pa4	-O8	-O9	72.3(3)
Pa1	-O1	-Pa2	110.7(3)	Pa3	-O9	-Pa4	106.2(2)
Pa1	-O1	-Pa3	108.5(2)	Pa3	-O9	-O8	72.8(3)
Pa1	-O1	-Pa4	109.5(3)	Pa4	-O9	-O8	70.9(3)
Pa2	-O1	-Pa3	109.5(3)	Pa2	-O10	-Pa4	105.6(3)
Pa2	-O1	-Pa4	109.2(2)	Pa2	-O10	-O11	71.4(4)
Pa3	-O1	-Pa4	109.4(3)	Pa4	-O10	-O11	70.7(4)
Pa1	-O2	-Pa3	105.8(3)	Pa2	-O11	-Pa4	106.4(3)
Pa1	-O2	-O3	72.9(3)	Pa2	-O11	-O10	71.6(3)
Pa3	-O2	-O3	70.8(3)	Pa4	-O11	-O10	72.4(3)
Pa1	-O3	-Pa3	105.5(2)	Pa2	-O12	-Pa3	106.8(2)
Pa1	-O3	-O2	70.4(3)	Pa2	-O12	-O13	70.2(3)
Pa3	-O3	-O2	72.6(4)	Pa3	-O12	-O13	73.2(3)
Pa1	-O4	-Pa2	106.6(2)	Pa2	-O13	-Pa3	106.8(2)
Pa1	-O4	-O5	75.5(4)	Pa2	-O13	-O12	72.9(4)
Pa2	-O4	-O5	69.5(4)	Pa3	-O13	-O12	70.4(3)

Translation of Symmetry Codes to Equivalent Positions

a = x,3/2-y,-1/2+z   b = x,3/2-y,1/2+z c = x,-1+y,z d = x,1+y,z e = x,1/2-y,-1/2+z

f = x,1/2-y,1/2+z g = 1-x,1-y,1-z h = -x,1-y,1-z
